# Synthesis, Characterization, and Biological Evaluation of Novel [M(η^6^-arene)_2_]^+^ (M = Re, ^99m^Tc) Hosted Terpyridines and Copper Complexes Thereof

**DOI:** 10.1021/acs.inorgchem.4c03018

**Published:** 2024-09-16

**Authors:** Joshua Csucker, Matthieu Scarpi-Luttenauer, Pierre Mesdom, Samia Hidalgo, Olivier Blacque, Gilles Gasser, Roger Alberto

**Affiliations:** † University of Zurich, Department of Chemistry, Winterthurerstrasse 190, 8057 Zürich, Switzerland; ‡ Chimie ParisTech, PSL University, CNRS, Institute of Chemistry for Life and Health Sciences, Laboratory for Inorganic Chemistry, 11, Rue Pierre et Marie Curie, F-75005 Paris, France; § Université de Paris, Institut de Physique du Globe de Paris, CNRS, F-75005 Paris, France

## Abstract

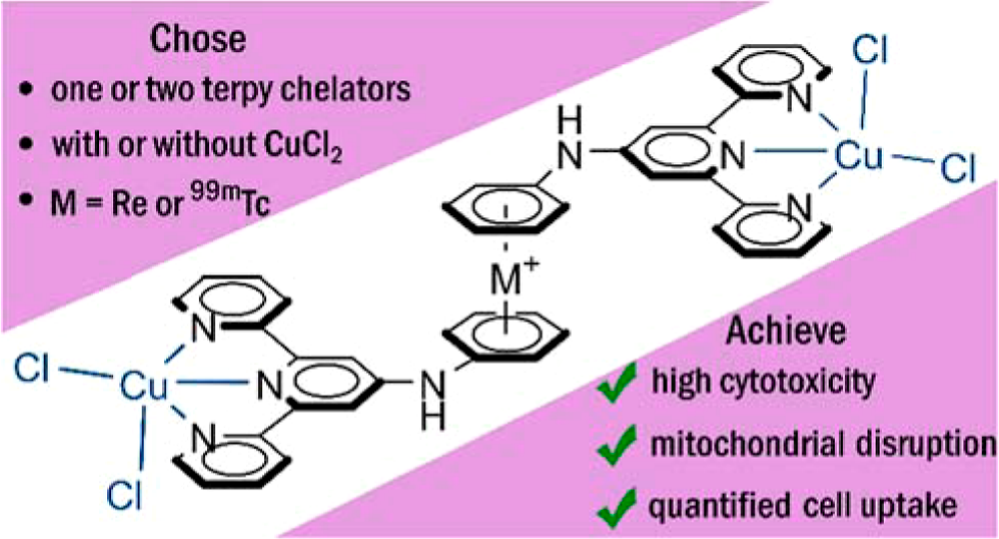

We report the synthesis, characterization, and in vitro biological activities of [Re(η^6^-arene)_2_]^+^-terpyridine conjugates and their Cu^II^ complexes. The terpyridine (terpy) chelators were attached to the [Re(η^6^-arene)_2_]^+^ scaffold via secondary amine linkers allowing for heteroleptic mono- and homoleptic bis-terpyridine-substituted chelators. Complexation with CuCl_2_ afforded the respective square pyramidal [Cu(terpy)Cl_2_] complexes hosted on the [Re(η^6^-arene)_2_]^+^ scaffold. The chelator conjugates and their respective complexes were found to be remarkably cytotoxic against malignant HT29 and A549 human cancer cell lines in vitro with IC_50_ values in the low micromolar range. Mitochondrial respiration disruption was identified as a possible mode of action of these novel drug candidates. Crucially, the [Re(η^6^-arene)_2_]^+^ hosts delivered water solubility of the otherwise insoluble [Cu(terpy)Cl_2_] motif. Importantly, the homoleptic [^99m^Tc(η^6^-arene)_2_]^+^-terpyridine conjugate is available in a single step, which enables the presented system to be used as a theranostic approach to modern medicinal inorganic chemistry.

## Abstract 2

We report the synthesis, characterization, and in vitro biological activities of [Re(η^6^-arene)_2_]^+^-terpyridine conjugates and their Cu^II^ complexes.

## Introduction

Modern medicinal chemistry is continuously exploring the chemical space in search of new bioactive motifs. Transition metal complexes have long been used in this context to develop either *de novo* drugs or expand existing lead structures. Prime examples thereof are cisplatin^1−3^ and ferroquine,^4−6^ offering reliable treatment options against cancer and malaria, respectively. Following their success, a plethora of metal- and specifically ferrocene-based drug candidates have been developed.^7^

[M(η^6^-arene)_2_]^+^ (M = Re, ^99m^Tc) sandwich complexes are isoelectronic to ferrocene. First reported by Fischer in the 1950s,^8,9^ this class of sandwich complexes was largely undeveloped for almost 60 years. Its chemistry recently experienced a renaissance after improved starting material syntheses were found.^10−15^ The [M(η^6^-arene)_2_]^+^ motif is highly stable under physiological conditions, water-soluble, and redox inert at a large potential window.^16^ Thus, it was quickly evaluated as a bioorganometallic scaffold.^11,12^ For example, incorporating rhenium bis-arenes into the Hoechst dyes core structure retained their DNA minor groove binding abilities.^17^ Coordinating the arenes in a plethora of approved pharmaceuticals such as lidocaine, hexestrol, melatonin, and paracetamol demonstrated that a large number of arenes available in modern pharmacopeia are suitable ligands for this compound class.^18^ Recently, [Re(η^6^-pseudo-erlotinib)_2_]^+^ was shown to have comparable biological activities to the parent small-molecule kinase inhibitor (pseudo)erlotinib.^19^ Together with its ^99m^Tc homologue, a true matched pair was obtained for theranostic investigations.

Given the above factors, it would be desirable to conjugate [M(η^6^-arene)_2_]^+^ with known cytotoxic units for the development of *de novo* drug candidates. For this purpose, we identified polypyridyl complexes with copper as attractive model bioinorganic motifs.^20^ Therein, terpyridine (terpy) has emerged as a versatile ligand for a plethora of [Cu(terpy)L_2_]-type complexes, where the overall charge depends on the supporting ligands L. Many cationic derivatives thereof are highly potent anticancer drug candidates.^20^ Similarly, [Cu(terpy)_2_]^2+^ and 4′-derivatives thereof were recently also shown to be decently cytotoxic in vitro.^21^ Mitochondrial respiration inhibition is thought to be a primary mode of action of copper polypyridyl complexes and specifically of square pyramidal [Cu(terpy)L_2_] complexes.^22,23^ Additionally, Choroba et al. recently prepared and studied dichloro-μ_2_-bridged [Cu_2_Cl_2_(terpy)_2_]^2+^ complexes, which cleave pDNA and show high in vitro cytotoxicity.^24^

A proof of principle of combining a sandwich motif with the [Cu(terpy)L_2_] motif for bioinorganic investigations was demonstrated by Deka and co-workers.^23^ Ferrocene was attached to [Cu(terpy)(acac)]^+^ (acac = acetylacetonate) structures at the 4′-position of the terpy. Doing so increased the potency of the copper complexes versus compounds featuring organic substituents.

Combining the diverse chemistry of [M(η^6^-arene)_2_]^+^ (M = Re, ^99m^Tc) with highly potent copper terpyridine complexes is a rational extension of those previous results. This appears particularly attractive in light of the fact that the [M(η^6^-arene)_2_]^+^ motif may enable water solubility of otherwise little soluble and therefore underdeveloped [Cu(terpy)Cl_2_] complexes. An additional interesting feature is the use of potential ^99m^Tc conjugates for imaging purposes. This study presents the synthesis and in vitro biological evaluation of such conjugates.

## Results and Discussion

### Re Compounds

**Scheme 1:**
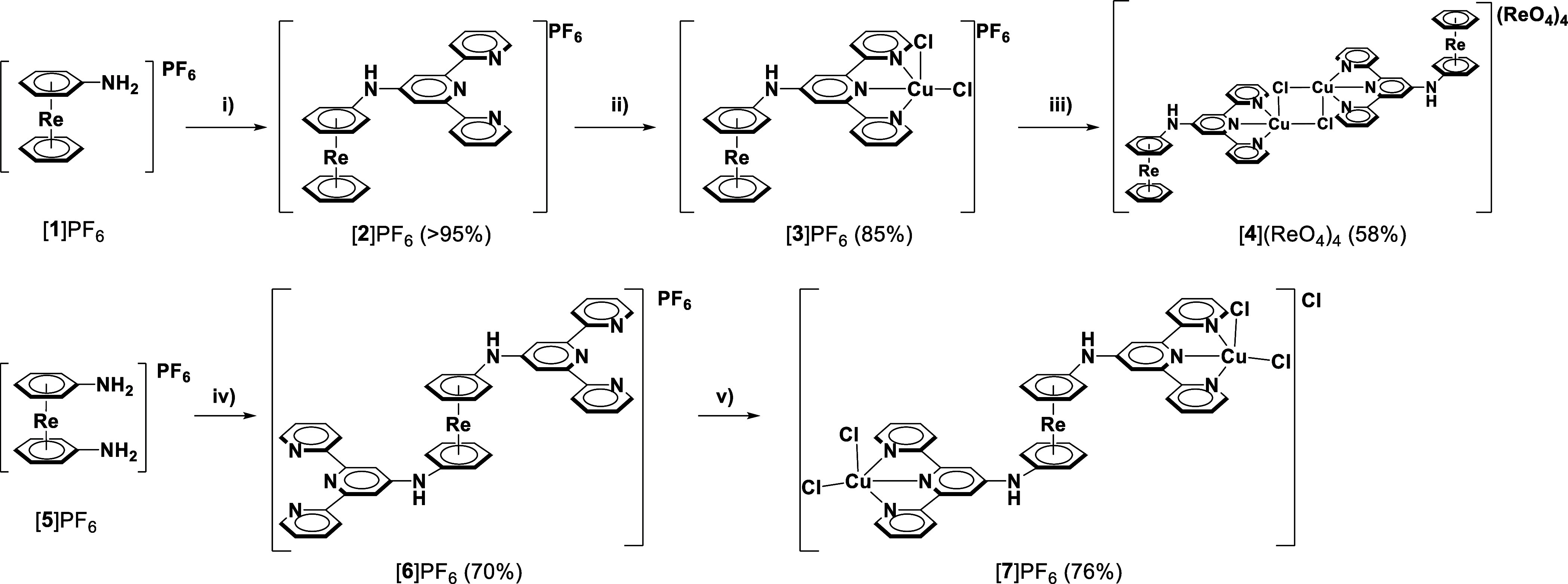
Synthesis of [Re(η^6^-Arene)_2_]^+^-Terpyridine Conjugate Chelators ([**2**]^+^ and [**2**]^+^) and Their Copper Complexes ([**3**]^+^, [**4**]^+^, and **[7]**^**+**^) Conditions: (i) 4-chloroterpyridine, JohnPhos, [Pd_2_(dba)_3_], KOtBu, 10:1 1,4-dioxane/THF, 100 °C, 3.5 h; (ii) CuCl_2_, MeOH, 25 °C, 1 h; (iii) excess KPF_6_ in MeOH/H_2_O, anion exchange over DOWEX 1:2 200–400 anion exchange resin; (iv) 4′-chloroterpyridine, JohnPhos, [Pd_2_(dba)_3_], KOtBu, 10:1 1,4-dioxane/THF, 100 °C, 14.5 h; (v) CuCl_2_, MeOH, 25 °C, 1 h.

We chose to conjugate terpyridine chelators to the [M(η^6^-arene)_2_]^+^ (M = Re, ^99m^Tc) motif via a secondary amine linker. The respective starting materials [Re(η^6^-aniline)(η^6^-C_6_H_6_)]^+^ ([**1**]^+^) and [Re(η^6^-aniline)_2_]^+^ ([**5**]^+^) are available through previously reported procedures.^14,18^ Palladium-assisted Buchwald–Hartwig-type cross coupling between [**1**]^+^ and 4′-chloroterpyridine led to the monosubstituted complex [**2**]PF_6_ in excellent isolated yields (>95%). JohnPhos as the supporting ligand and [Pd_2_(dba)_3_] as the precatalyst lead to optimal reaction outcomes. Coordination of CuCl_2_ to [**2**]PF_6_ in MeOH provided [**3**]PF_6_ (85%). Treatment with excess KPF_6_ followed by ion exchange chromatography over DOWEX resin removed one axial chloride ligand from [**3**]^+^ and afforded complex [**4**](ReO_4_)_4_ (58%, Scheme 1).

Exchange of the chloro ligands in [**3**]^+^ with coordinating solvents such as DMSO, acetonitrile, or methanol was not observed at room temperature. Water slowly displaced the axial chloro ligand over the course of 1 week during crystallization attempts. A single crystal structure thereof is presented in the Supporting Information (Figure S21 and Tables S7–S8). Ligand exchange was suppressed by the addition of small amounts of NaCl. Accordingly, the biological activity of [**3**]^+^ was expected to be influenced by the hyperlocal chloride concentration in the cell. Similarly, [**4**]^4+^ may exist in an equilibrium with [**3**]^+^ in vitro or in vivo and vice versa.

Bis-substituted [Re(η^6^-arene)_2_]^+^-terpy conjugates were obtained following the same synthetic strategy as executed with monosubstituted analogues. Buchwald–Hartwig cross coupling between [**5**]PF_6_ and 4′-chloroterpyridine formed [**6**]PF_6_ (70%). Coordination to CuCl_2_ proceeded smoothly, delivering [**7**]Cl in 76% isolated yield as a green solid. Removal of the axial chloro ligands in [**7**]Cl was omitted. Doing so would most likely lead to oligo/polymeric structures, and their biological activities can hardly be compared with the well-characterized mono- or bimolecular analogues [**3**]^+^ or [**4**]^+^.

All compounds studied in this work are fully characterized by standard analytical methods and single-crystal XRD analyses. NMR spectroscopy of the copper complexes was not feasible due to their strong paramagnetism. Single-crystal XRD analyses unambiguously confirmed the chemical structures of [**2**]^+^**–**[**4**]^4+^ and [**6**]^+^. Independently, [**1**]H(PF_6_)_2_ and [**2**]PF_6_ crystallized in the monoclinic space group *P*2_1_/*c* as yellow crystals. Protonation of the terpyridine motif in [**1**]^+^ forced the nitrogen atoms of the chelator to face one another, while the free base structure of [**6**]^+^ showed alternating arrangement of the pyridines to minimize steric interactions of the chelator. The bridging nitrogen atom connecting [Re(η^6^-arene)]^+^ and terpyridine subdomains in both [**1**]^+^ and [**6**]^+^ is sp^2^ hybridized according to its bond angles. Both the C7–N1–C13 (124.2(5)°) angle in [**2**]^+^ and the corresponding C–N–C angles in [**6**]^+^ (129.6(5)°/129.2(5)° are widened versus the ideal 120° due to steric repulsion of the two subunits. Green needles of [**3**]Cl·(H_2_O)_2_ were obtained by slow evaporation of THF into a solution of [**3**]PF_6_ in 1:1 sat. aq NaCl/CH_3_CN. Attempts of crystallizing [**3**]PF_6_ directly formed amorphous powders unsuitable for analysis. The [Cu(terpy)Cl_2_] motif in [**3**]Cl·(H_2_O)_2_ was square pyramidal. Axial distortion was observed with the copper center above the plane of the terpyridine. Chelation of the terpyridine forced angle sharpening along the N2–Cu1–N4 (156.30(17)°) axis. The N3–Cu1–Cl1 (97.76(13)°) and Cl1–Cu1–Cu2 (103.82(6)°) angles were noticeably larger than the ideal 90°. Furthermore, the axial Cl1–Cu1 (2.5176(15) Å) bond was elongated versus the equatorial Cl2–Cu1 (2.2625(15)Å) These data were in agreement with an in-depth discussion of Kutoglu et al. on [Cu(terpy)X_2_] (Y = I^–^, NCS^–^) complexes.^25^

**Figure 1:**
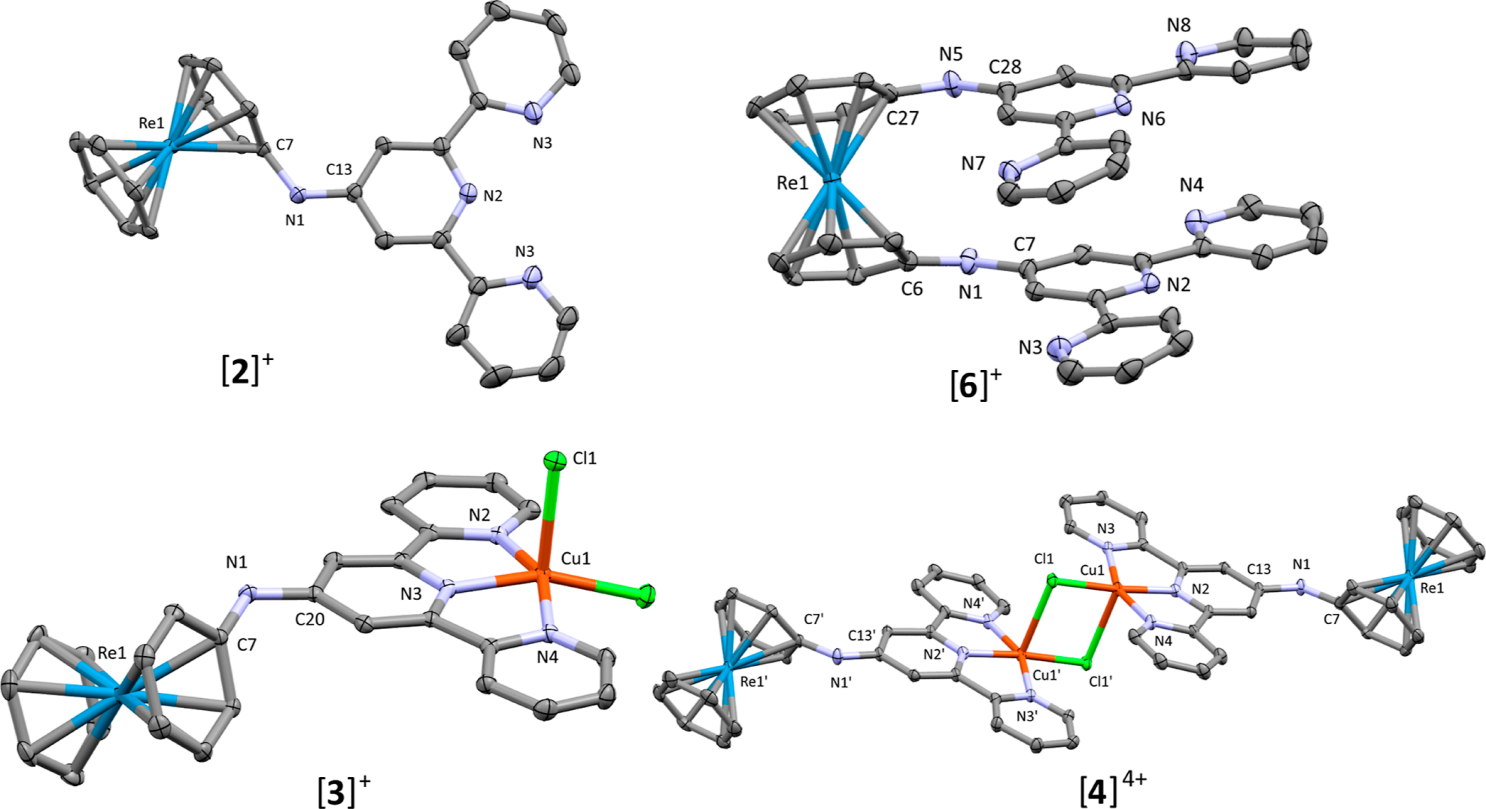
Ellipsoid displacement plot of [**2**]^+^ of the crystal structure [**2**]H(PF_6_)_2_ (top left), [**6**]^+^ of the crystal structure [**6**]PF_6_ (top right), [**3**]^+^ of the crystal structure [**3**]Cl·(H_2_O)_2_ (bottom left), and [**4**]^4+^ of the crystal structure [**4**](ReO_4_)_4_·(H_2_O)_2_ (bottom right). Thermal ellipsoids represent 50% probability. Hydrogen atoms and counterions are removed for clarity. Selected bond lengths and angles are provided in the Supporting Information (Figures S18–S22; Tables S2–S10).

The dimerized compound [**4**](ReO_4_)_4_ crystallized as dark green needles in the triclinic space group *P*1̅. Isostructural examples based on native terpy were described by Rojo et al.^24,26,27^ Individually, the [Cu(terpy)Cl_2_] motifs were similarly distorted as those in the monomeric [**3**]^+^. Further axial elongation of the Cu1–Cl1′ (2.7123(17)°) bond was in line with observations of Rojo et al.^26,27^ Ellipsoid displacement plots of the cations [**2**]^+^, [**3**]^+^, [**4**]^+^, and [**6**]^+^ are shown in Figure 1.

### ^99m^Tc Compounds

**Scheme 2:**
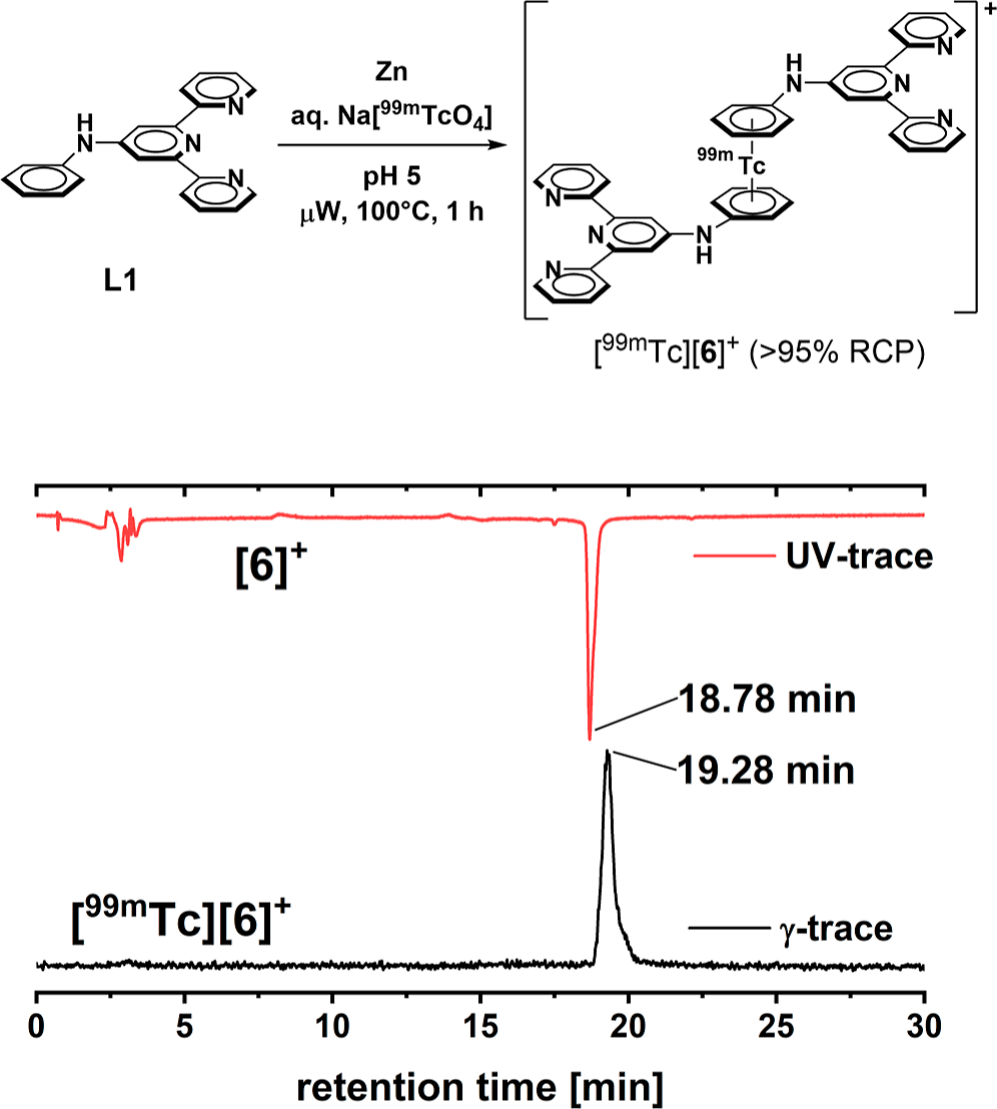
Radiolabeling of **L1** with aq Na[^99m^TcO_4_] Afforded [^99m^Tc][**6**]^+^ in a Single Step in >95% RCP (Top) after Purification HPLC of the UV and γ-traces of [**6**]^+^ and [^99m^Tc][**6**]^+^, respectively (bottom).

For quantitative and qualitative imaging purposes, the preparation of terpy-substituted [^99m^Tc(η^6^-arene)_2_]^+^ homologues of [**6**]^+^ is of great interest. We have reported on the direct preparation of [^99m^Tc(η^6^-arene)_2_]^+^ complexes from aqueous [^99m^TcO_4_]^−^ and the corresponding arenes in the past.^14,15,18,19^ The use of arene **L1** for the preparation of [^99m^Tc][**6**]^+^ was described by Constable.^28^ It was obtained by nucleophilic aromatic substitution between aniline and 4’–chloroterpyridine. Reacting **L1** with aq Na[^99m^TcO_4_] under slightly acidic conditions gave [^99m^Tc][**6**]^+^ as the dominant ^99m^Tc species with *R*_t_ = 19.3 min in 75% radiochemical purity (RCP). Recovery of activity was 60%. Semipreparative purification from the crude labeling solution in the region of interest increased the RCP to >95% (Scheme 2, top).

[^99m^Tc][**6**]^+^ is available as a straightforward reaction. It can be conveniently separated from excess free ligand to obtain it in pure form. Competition between arene coordination and terpy chelation to technetium was suppressed as in the acidic medium, the nitrogen atoms of the terpy motif are protonated. Chromatographic coinjection with the corresponding rhenium standard [**6**]^+^ was performed for authenticity confirmation of the ^99m^Tc species (Scheme 2, bottom). The UV trace showed a single peak for [**6**]^+^ at *R*_t_ = 18.78 min, with the corresponding γ-signal for [^99m^Tc][**6**]^+^ at *R*_t_ = 19.28 min. The difference of +0.5 min between both signals is in agreement with the dead volume separating the two detectors.^15^ With [^99m^Tc][**6**]^+^ available directly from aqueous Na[^99m^TcO_4_], it is principally possible to apply [M(η^6^-arene)_2_]^+^-terpy conjugates (M = Re, ^99m^Tc) in theranostic medicinal chemistry. The matched pair between radioactive 99m-technetium and stable rhenium species allows for continued (radio)pharmaceutical assessment of the ligand system at hand.

### Solubility Studies

Water solubility of chelators and copper complexes described previously is crucial for biological assessments. All respective PF_6_^–^ salts are insoluble in water. Protonation of chelators [**2**]^+^ and [**6**]^+^ with trifluoroacetic acid generates nonhygroscopic water-soluble complex salts. The same cannot be accomplished with the copper complexes, as the respective TFA salts are hygroscopic. The Cl^–^ salts were equally problematic for [**3**]^+^ and [**4**]^4+^. Only [**7**]Cl is both nonhygroscopic and water-soluble. We found that [ReO_4_]^−^ salts of [**3**]^+^ and [**4**]^4+^ were well suited for biological evaluation as they were isolated as green dry powders after freeze-drying.

### Cytotoxicity Studies on a 2D Cell Model

First, the cytotoxic activity of the compounds was investigated in different cancerous and noncancerous cell lines, namely, HT29 (human colon adenocarcinoma), A549 (human lung adenocarcinoma), and RPE-1 (human retinal epithelium). Cisplatin was used as a positive control. Cells were first incubated for 48 h with the complexes before cell viability was measured with a colorimetric resazurin assay. IC_50_ data are given in Table 1.

**Table 1:** IC_50_ (μM) Values ± SEM for the Targeted Compounds in Three Different Cell Lines^a^

	HT29	A549	RPE-1
cisplatin	7.84 ± 0.57	10.9 ± 0.17	22.6 ± 2.74
[**2**]TFA	1.6 ± 0.08	9.0 ± 0.85	2.9 ± 1.16
[**3**]ReO_4_	7.4 ± 3.2	13.8 ± 1.8	8.13 ± 1.10
[**4**](ReO_4_)_4_	4.2 ± 0.37	6.3 ± 0.54	10.3 ± 0.51
[**6**]TFA	4.3 ± 0.76	2.8 ± 0.19	9.72 ± 0.52
[**7**]Cl	0.92 ± 0.09	6.7 ± 0.38	10.0 ± 1.3
CuCl_2_	>100	>100	>100
(NH_4_)[ReO_4_]	>100	>100	>100

a Representative data from three independent experiments are shown. CuCl_2_ and (NH_4_)[ReO_4_] were tested as negative controls.

All compounds show significant in vitro toxicities in a range from 1 to 10 μM against all cell lines. The IC_50_ values reported are similar to or lower than those of cisplatin. It should be stressed that for [**6**]TFA, [**4**]ReO_4_, and [**7**]Cl, the highest IC_50_ values are found when the compounds were incubated in the noncancerous cell line RPE-1. Worthy of note, for each cell line, the lowest IC_50_ is found for different compounds. With the HT29 cell line, the lowest IC_50_ value is found when the cells are incubated with [**7**]Cl, with a remarkable value of 0.92 ± 0.09 μM.

Concerning the A549 cell line, the lowest IC_50_ value is found when the cells are incubated with [**6**]TFA (IC_50_ = 2.8 ± 0.19 μM). For the RPE-1 cell line, the lowest IC_50_ value is found after incubation with [**2**]TFA (2.9 ± 1.16 μM).

Compounds [**2**]TFA and [**6**]TFA, bearing free ligands, show similar activities compared to the Cu(I) complexes. Cytotoxic activities of polypyridyl ligands have been investigated in the past, notably, by Dwyer et al.,^29^ who showed that 1,10-phenanthroline can be toxic by sequestrating metals essential for cellular metabolic activity. It is therefore not surprising to observe the toxicity of the two aforementioned compounds.

### Evaluation of the Mitochondrial Respiration Disruption

**Figure 2:**
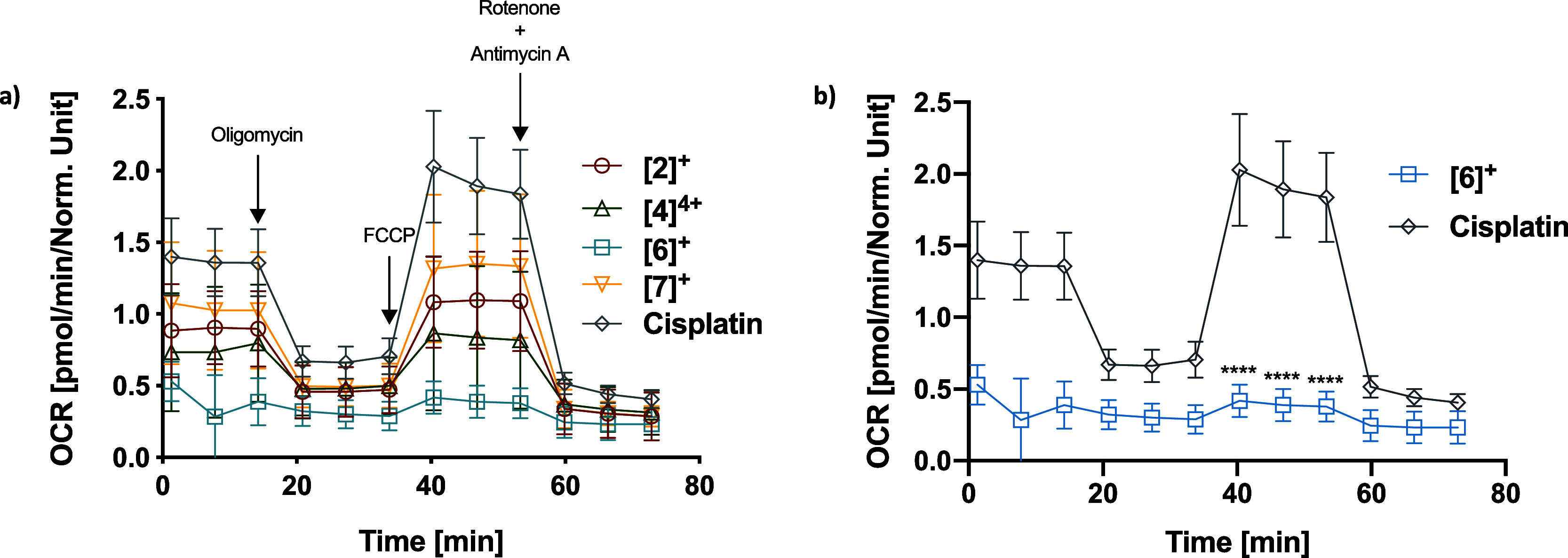
(a) Mito stress test of the OCR profile in HT29 cells after 24 h treatment. Oligomycin (inhibitor of ATP synthase), FFCP (uncoupling agent), antimycin A (complex III inhibitor), and rotenone (complex I inhibitor) were sequentially added. Their addition times are shown by arrows. The values are expressed as a mean ± standard error median of three repeats. (b) Comparison of the Mito stress test of cisplatin (green) and [**6**]TFA (yellow). ****P < 0.0001.

Having the cytotoxicity profile of all of our compounds on three different cell lines in hand, the mechanism of action of the complexes was evaluated. First, their ability to disrupt mitochondrial respiration was assessed against HT29 cells using a SeaHorse XF96 apparatus (Agilent). Cisplatin, which is known not to disrupt mitochondrial homogeneity and functionality, was used as a control. All the compounds were tested at their IC_25_ for 4 h. The oxygen consumption rate (OCR) of the mitochondria was then measured after sequential addition of oligomycin (ATP synthase inhibitor), FCCP (carbonylcyanide-*p*-trifluoromethoxyphenylhydrazone, a mitochondrial membrane uncoupling agent), and rotenone + antimycin A (mitochondrial respiratory complexes I and III inhibitors, respectively) (Figure 2a).^30^

Overall, the complexes studied show a reduced oxygen consumption compared to cisplatin. This result illustrates the effect of all the complexes on mitochondrial respiration both for basal respiration (meaning that a concentration as low as the IC_25_ value already affects the cellular respiration) and the maximal respiratory capacity (meaning that the respiratory chain capacity is disrupted). For [**6**]TFA in particular, nearly no oxygen consumption is observed during the time of the experiment (Figure 2b), independently of the addition of mitochondrial respiration modifiers, suggesting a higher (or almost total) disruption effect on the mitochondrial chain activity and capacity.

### Cellular Localization Study by Inductively Coupled Plasma Mass Spectrometry

The localization of the complexes was evaluated by inductively coupled plasma mass spectrometry (ICP-MS). HT29 cells were incubated with the compounds at 10 μM for 4 h.

**Figure 3:**
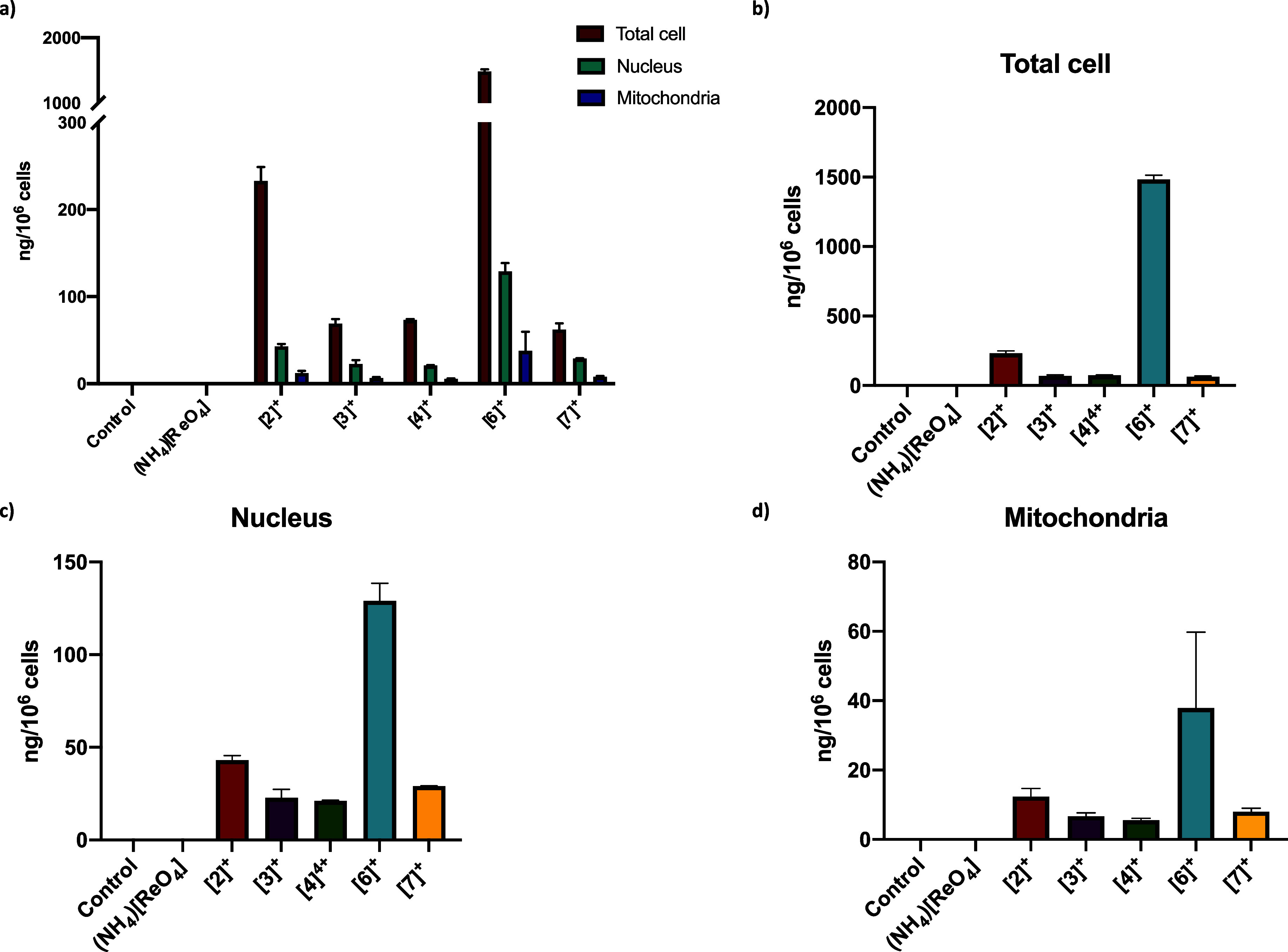
(a) Subcellular distribution of rhenium upon incubation of the compounds (10 μM) in HT29 cells for 4 h determined by ICP-MS. Untreated cells have been used as control experiments. The *y*-axis is cut between 300 and 1000 ng/10^6^ cells for clarity. (b) Quantification of the rhenium in the total cell content. (c) Quantification of the rhenium in the nucleus. (d) Quantification of rhenium in the mitochondria. The values are expressed as a mean ± SEM of two repeats. Numerical values are given in the electronic Supporting Information in Table S1.

Three separate batches of cells were then fractionated to isolate the total cell, nucleus, and mitochondria contents separately. The quantity of rhenium present in each batch for every compound was measured by ICP-MS, and the results are shown in Figure 3.

Overall, the quantification obtained for the total cell shows that the polypyridyl rhenium complexes exhibit better accumulation than (NH_4_)[ReO_4_], probably due to the higher lipophilicity of the compounds (Figure 3b). In particular, high levels of cellular rhenium uptake are observed for [**6**]TFA (1482 ng/10^6^ cells). The results obtained for the accumulation of rhenium in the mitochondria and the nucleus show the same pattern than the total cell analysis (Figure 3c,d). Higher levels of [**6**]TFA are found in the nucleus and mitochondria of the cells, which is consistent with the hypothesis that the compounds do not target a specific organelle.

The high levels of [**6**]TFA found in the mitochondria compared to the other complexes studied correlates to the high mitochondrial respiration disruption observed when cells are incubated with [**6**]TFA. Interestingly, although [**6**]TFA accumulates the most in HT29 cells, [**7**]Cl remains more cytotoxic than [**6**]TFA (IC_50_ values of 0.92 ± 0.09 μM and 4.3 ± 0.76 μM, respectively), indicating that the presence of Cu(II) enhances the cytotoxic efficacy of the complexes.

## Conclusions

In summary, this study presents a synthetic approach to attach terpyridines to [M(η^6^-arene)_2_]^+^ (M = Re, ^99m^Tc) complexes via a secondary amine linker. Doing so afforded mono- and bis-substituted chelators [**2**]^+^ and [**6**]^+^, which coordinated strongly with Cu^II^. These chelators and their copper complexes are highly cytotoxic in vitro against the cancerous HT29 and A549 cell lines. Their IC_50_ values are significantly higher than those of cisplatin, in the case of [**7**]Cl in HT29 by as much as 10-fold. Selectivity of malignant versus healthy cells (RPE1) was the highest for [**7**]Cl in HT29 and [**6]**TFA in A459. Concerning [**6**]TFA, mitochondrial respiration disruption was identified as a primary mode of action. No clear effect of having one versus two biologically active functional groups attached to the [Re(η^6^-arene)_2_]^+^ base scaffold was observed. The ^99m^Tc compound may serve as a radiopharmacological probe for further investigations, while continued biological evaluation can be achieved with the rhenium compounds.

The synthetic strategy presented may be extended to other chelators of interest with minimal synthetic adaptation. As terpyridine chelates a plethora of transition metals, generalization of this system appears reasonable.

## Experimental Section

This section discusses the general procedures for instrumentations and techniques and common starting materials. Individual reaction schemes are provided in Chapter 2 of the Supporting Information.

**[2]**PF_6_: A 50 mL Schlenk round-bottom flask was charged with **[1]**PF_6_ (70 mg, 140 μmol, 1.0 equiv), 4′-chloroterpyridine (56 mg, 209 μmol, 1.5 equiv), JohnPhos (4 mg 0.1 equiv), Pd_2_dba_3_ (13 mg, 0.1 equiv), and KOtBu (23 mg, 209 μmol, 1.5 equiv). The solids were dissolved in dry THF (5 mL) and 1,4-dioxane (20 mL). The resulting orange mixture was stirred at 100 °C for 3.5 h. The crude reaction mixture was cooled to r.t. and diluted with H_2_O (40 mL). The brown suspension was washed with Et_2_O (3 × 20 mL). The aq phase was acidified with TFA (1 mL), which changed the color from pale brown to intense yellow. The aq phase was passed over a SEP-PAK plus C18 SPE cartridge. NH_4_PF_6_ was added to the filtrate, and the pH was adjusted to 10 by the addition of sat. aq NaHCO_3_. The aq phase was extracted with CH_2_Cl_2_ (3 × 20 mL). The combined org. layers were dried over MgSO_4_, and the solvent was removed in vacuo. The solids were lyophilized to provide the title compound **[2]**PF_6_ (103 mg, 140 μmol, > 95%) as a pale-yellow solid. The material may also be purified by preparative HPLC if conversion of the starting material is incomplete or the crude product is of insufficient purity. The pH of fractions containing the title compounds must be adjusted to 10 prior to anion exchange as the HTFA salt of **[2]**^**+**^ is readily H_2_O soluble.

^**1**^**H NMR** (CDCl_3_, 400 MHz): δ (ppm) 8.73 (*ddd*, *J* = 4.7, 1.8, 0.9 Hz, 2 arom. CH, H_4_), 8.66 (*dt*, *J* = 7.9, 1.1 Hz, 2 arom. CH, H_7_); 8.12 (*s*, 2 arom. CH, H_9_); 7.97 (*td*, *J* = 7.7, 1.8 Hz, 2 arom. CH, H_5_); 7.47 (*ddd*, *J* = 7.5, 4.8, 1.2 Hz, 2 arom CH, H_6_); 7.22 (*s*, 1 NH); 6.40 (*d*, *J* = 6.0 Hz, 2 arom CH, H_13_), 6.09 (*t, J* = 5.9 Hz, 2 arom. CH, H_12_); 6.04 (s, 6 arom. CH, H_10_); 5.84 (t, *J* = 5.2 Hz, 1 arom. CH, H_11_). ^**13**^**C NMR** (CDCl_3_, 125 MHz): δ (ppm) 156.4 (2 arom. C_q_, C_1_); 155.4 (2 arom. C_q_, C_2_); 150.4 (1 arom. C_q_, C_3_); 149.2 (2 arom. CH, C_4_); 137.3 (2 arom. CH, C_5_); 124.4 (2 arom. CH, C_6_); 120.9 (2 arom. CH, C_7_); 108.3 (1 arom. C_q_, C_8_); 108.2 (2 arom. CH, C_9_) 76.5 (6 arom. CH, C_10_) 75.8 (1 arom. CH, C_11_); 74.8 (2 arom. CH, C_2_); 69.7 (2 arom. CH, C_2_) (Signal assignment corresponds to atom numbering depicted in Scheme S1). **FT-IR** (KBr) ν[cm^–1^]: 3366*w*, 3145*w*, 3090*w*, 1638*m*, 1584*m*, 1519*m*, 1452*m*, 1249*m*, 995*w*, 837*s*, 740*m*. **HR-ESI-MS**: [M]^+^ = [C_27_H_22_N_4_Re]^+^; calc.: 589.13965 *m*/*z*, found: 589.14001 *m*/*z* (0.62 Δppm); [M + H]^2+^ = [C_27_H_23_N_4_Re]^2+^; calcd: 295.07346 *m*/*z*, found: 295.07361 *m*/*z* (0.51 Δppm).

**[3]**PF_6_: A 50 mL Schlenk round-bottom flask was charged with MeOH (20 mL) and **[2]**PF_6_ (9.9 mg, 13.6 μmol, 1.0 equiv). The off-yellow solution was degassed under N_2_ flow for 10 min. CuCl_2_ (5.5 mg, 40.9 μmol, 3.0 equiv) was dissolved in degassed MeOH (5 mL) and added dropwise to the reaction solution. The color changed to deep green. The reaction mixture was stirred for 1 h during which it became cloudy. The reaction mixture was concentrated to one-third of the original volume, and the green solids were collected by centrifugation. The solids were washed with MeOH (10 mL) and collected by filtration. The title compound **[3]**PF_6_ (10.1 mg, 11.6 μmol, 85%) was isolated as a green solid. ReO_4_^–^ was achieved by passing a solution of **[3]**PF_6_ in DMSO/0.01 M aq NaRe_4_ (5 mL) over DOWEX 1:2 200–400 anion exchange resin, which was equilibrated with a 0.01 M aq NaReO_4_ solution. The filtrate was collected and lyophilized. Residual NaReO_4_ was removed by extraction of the solids with MeOH (2 × 3 mL). The solvent was removed in vacuo.

**FT-IR** (KBr) ν[cm^–1^]: 3225*w*, 3049*w*, 2925*w*, 1615*m*, 1536*m*, 1476*m*, 1434*m*, 1385*m*, 1286*w*, 1254*m*, 1033*w*, 840*s*, 791*m*. **HR-ESI-MS**: [M–Cl]^2+^ = [C_27_H_22_ClCuN_4_Re]^+^; calcd: 343.51803 *m*/*z*, found: 343.51863 *m*/*z* (−0.43 Δppm); [M–HCl–Cl]^+^ = [C_27_H_21_ClCuN_4_Re]^+^; calcd: 686.02923 *m*/*z*, found: 686.02869 *m*/*z* (−1.36 Δppm); [M–HCl–H + COOH]^+^ = [C_28_H_22_O_2_N_4_CuRe]^+^; calcd: 694.05628 *m*/*z*, found: 694.05628 *m*/*z* (0.55 Δppm).

**[3]**ReO_4_: **ICP-MS**: Determination of w/w % content of Cu for C_27_H_22_Cl_2_CuN_4_O_4_Re_2_: found: 6.65 w/w %, Cu calculated 6.53 w/w %. Relative error: 1.8%. **EA:** 38.04% C, 3.61% H, 4.42% N.

**[4]**(ReO_4_)_4_: A centrifuge vial was charged with **[3]**PF_6_ (10.0 mg, 11.5 μmol, 1.0 equiv), CH_3_CN (3 mL), and H_2_O (4 mL). Excess NH_4_PF_6_ (50 mg) was added to the green solution. After 5 min, a dark green precipitate formed. The precipitate was collected and dissolved in DMSO (0.5 mL) and 0.01 M aq NaReO_4_ (5 mL). The green solution was passed over DOWEX 1:2 200–400 anion exchange resin, which was equilibrated with 0.01 M aq NaReO_4_ solution The filtrate was collected and lyophilized. Residual NaReO_4_ was removed by extraction of the solids with cold MeOH (2 × 3 mL). The solvent was removed in vacuo. The crude was recrystallized by slow evaporation of acetone into a solution of the analyte in DMSO. The title compound **[4]**(ReO_4_)_4_ (8.1 mg, 6.7 μmol, 58%) was isolated as long green needles.

FT-IR (KBr) ν[cm^–1^]: 3430s, 3058w, 1616s, 1543s, 1543m, 1474s, 1442m, 1374m, 1253w, 1030w, 793w. ICP-MS: determination of w/w % content of Cu for C_54_H_44_C_l2_Cu_2_N_8_O_16_Re_6_: found: 5.35 w/w %, Cu calculated 5.85 w/w %. Relative error: 8.5%.

**[6]**PF_6_: A 50 mL Schlenk round-bottom flask was charged with **[5]**PF_6_ (200 mg, 319 μmol, 1.0 equiv), 4′-Cl-terpy (233 mg, 816 μmol, 2.2 equiv), JohnPhos (21 mg, 63 μmol, 0.2 equiv), Pd_2_dba_3_ (56 mg, 63 μmol, 0.2 equiv), and KOtBu (90 mg, 8.16 μmol, 2.5 equiv). The solids were dissolved in dry THF (5 mL) and dry 1,4-dioxane (20 mL). The resulting orange mixture was stirred at 100 °C for 14.5 h. The solvent was removed in vacuo to give a brown solid residue. The crude reaction mixture was cooled to r.t. and diluted with H_2_O (40 mL). The brown suspension was washed with Et_2_O (3 × 20 mL). The aq phase was acidified with TFA (1 mL), which changed the color from pale brown to intensely yellow. The aq phase was passed over a SEP-PAK plus C18 SPE cartridge. NH_4_PF_6_ was added to the filtrate, and the pH was adjusted to 10 by addition of sat. aq NaHCO_3_. The aq phase was extracted with CH_2_Cl_2_ (3 × 20 mL). The combined org. layers were dried over MgSO_4_, and the solvent was removed in vacuo. The solids were lyophilized to provide the title compound **[6]**PF_6_ (265 g, 270 mmol, 70%) as a pale-yellow solid. The material may also be purified by preparative HPLC if conversion of the starting material is incomplete or the crude product is of insufficient purity. Fractions containing the title compound were combined in a separatory funnel. NH_4_PF_6_ (100 mg) was added, and the pH of the solution was adjusted to 9 by the addition of sat. NaHCO_3_ (50 mL). The aq phase was extracted into CH_2_Cl_2_ (2 × 50 mL). The organic layers were combined and dried over MgSO_4_. The solvent was removed in vacuo, and the title compound can be collected as an off-yellow solid after lyophilization.

^**1**^**H NMR** (CD_3_CN, 500 MHz): δ (ppm) 8.46 (*d*, *J* = 4.7 Hz, 4 arom. CH, H_4_); 8.28 (*d*, *J* = 7.9 Hz, 4 arom. CH, H_7_); 7.91 (*s*, 4 arom. CH, H_9_); 7.76 (*d*, *J* = 1.8 Hz, 1 arom. CH, H_5_); 7.25 (dd, *J* = 7.5, 4.7 Hz, 4 arom.CH, H_6_); 6.60 (*d*, *J* = 5.5 Hz, 4 arom. CH, H_12_); 6.05 (*t*, *J* = 5.4 Hz, 4 arom. CH, H_11_); 5.77 (*t*, *J* = 5.2 Hz, 2 arom. CH, H_10_). ^**13**^**C NMR** (CDCl_3_, 126 MHz): δ (ppm) 156.0 (4 arom. C_q_, C_1_); 155.2 (4 arom. C_q_, C_2_); 149.7 (2 arom. C_q_, C_3_); 148.8 (4 arom. CH, C_4_); 136.9 (4 arom. CH, C_5_); 123.9 (4 arom. CH, C_6_); 120.8 (1 4 arom. CH, C_7_); 108.7 (2 arom. C_q_, C_8_); 108.1 (4 arom. CH, C_9_); 75.5 (2 arom. CH, C_10_); 74.1 (4 arom. CH, C_11_); 69.7 (4 arom. CH, C_12_) (some quaternary carbons were not observed due to long relaxation; signal assignment corresponds to atom numbering depicted in Scheme S4 in the Supporting Information). **FT-IR** (KBr) ν[cm^–1^]: 3391*w*, 3098*w*, 1636*m*, 1585*m*, 1525*m*, 1455*m*, 1384*m*, 1202*w*, 836*s*, 741*w*. **HR-ESI-MS**: [M]^+^ = [C_42_H_32_N_8_Re]^+^; calcd: 835.23020 *m*/*z*, found: 835.22950 *m*/*z* (−0.83 Δppm).

[^99m^Tc]**[6]**^**+**^: Ligand **L1** (4 mg) and Zn° chips (14 mg) were loaded into a microwave vial. The vial was flushed with N_2_ for about 5 min. Generator eluate (1.8 GBq, 6 mL) was mixed with aq HCl (300 μL, 1 M), and 1 mL of this solution was added to the microwave vial. The vial was heated for 30 min at 100 °C and brought to r.t. The turbid solution (undissolved ligand) was syringe filtered (2 μm) to yield a clear solution with residual ligand the product. Labeling yields varied between 30 and 60%. Typical HPLC traces are given in Figure S17.

**[7]**Cl: CuCl_2_ (13 mg, 94 μmol, 5.0 equiv) was dissolved in MeOH (25 mL) in a 50 mL Schlenk round-bottom flask A solution of **[6]**PF_6_ (18.5 mg, 19 μmol, 1.0 equiv) in CH_3_CN (3 mL) was added. The off-yellow solution was added dropwise to the reaction solution. The color changed to deep green. The reaction mixture was stirred for 1 h during which it became cloudy. The crude reaction mixture was concentrated to 5 mL, and the green solids were collected by centrifugation to give **[7]**PF_6_ (18.5 mg, 15 μmol, 78%). Anion exchange was accomplished by dissolving crude **[7]**PF_6_ in DMSO (0.5 mL) and 0.01 M aq HCl (5 mL). The green solution was passed over DOWEX 1:2 200–400 anion exchange resin, which was equilibrated with 0.01 M aq HCl. The filtrate was collected and lyophilized twice. The title compound **[7]**Cl (16.3 mg, 14.3 μmol, 76%) was obtained as a fluffy green powder.

**FT-IR** (KBr) ν[cm^–1^]: 3436*m*, 3063*w*, 1619*s*, 1546*m*, 1521*m*, 1475*s*, 1451*s*, 1385*s*, 1254*w*, 1032*w*, and 788*w*. **HR-ESI-MS:** [M]^+^ = [C_42_H_32_Cl_4_Cu_2_N_8_Re]^+^; calcd: 1100.96480 *m*/*z*, found: 1100.96256 *m*/*z* (−0.50 Δppm); [M–Cl]^2+^ = [C_42_H_32_Cl_3_Cu_2_N_8_Re]^+^; calcd: 532.99770 *m*/*z*, found: 532.99670 *m*/*z* (−0.51 Δppm). **ICP-MS**: determination of w/w % content of Cu for C_42_H_32_Cl_5_Cu_2_N_8_Re: found: 11.41 w/w %, Cu calculated 11.16 w/w %. Relative error: 2.2%, **EA:** 36.13% C, 3.07% H, 5.78% N.

## Supporting Information Available

Detailed biological experiments, instrumentation, and methods; ^1^H NMR, ^13^C NMR, HSQC, and HMBC spectra of diamagnetic compounds; spectroscopic analyses; and crystallographic data (PDF)

## Abbreviations

dba: ((1*E*,4*E*)-1,5-diphenylpenta-1,4-dien-3-one)
JohnPhos: 2-(di-*tert*-butylphosphino)biphenyl
terpy: 2,2′:6′,2″-terpyridine
OCR: oxygen consumption rate
