# Supplementary material for: Synthesis, Characterization, and Biological Evaluation of Novel [M(η6-arene)2]+ (M = Re, 99mTc) Hosted Terpyridines and Copper Complexes Thereof
Source: Inorg Chem. 2024 Sep 16;63(39):18154–61. doi: 10.1021/acs.inorgchem.4c03018 (PMC11445722; doi:10.1021/acs.inorgchem.4c03018)
Supplement: Supplementary file 1 — ic4c03018_si_001.pdf [file ic4c03018_si_001.pdf]

Supporting information

Synthesis, Characterization and Biological Evaluation of Novel $[M(\eta^6\text{-arene})_2]^+$ (M = Re, ^{99m}Tc) Hosted Terpyridines and Copper Complexes Thereof

Joshua Csucker,[a] Matthieu Scarpi-Luttenauer,[b] Pierre Mesdom,[b] Samia Hidalgo,[c] Olivier Blacque,[a] Gilles Gasser,[a] Roger Alberto[a]

a) University of Zurich, Department of Chemistry, Winterthurerstrasse 190, 8057 Zürich, Switzerland.

b) Chimie ParisTech, PSL University, CNRS, Institute of Chemistry for Life and Health Sciences, Laboratory for Inorganic Chemical Biology, 11, rue Pierre et Marie Curie, F-75005 Paris, France.

c) Université de Paris, Institut de physique du globe de Paris, CNRS, F-75005 Paris, France

Table of Contents

1. Cellular Re Concentration Determined by ICP-MS 2

2. Synthetic Procedures 2

General Materials and Methods 2

Reaction Schemes 6

3. NMR Data 8

4. FT-IR Data 11

5. HR-ESI-MS Data 13

6. HPLC UV/Vis and γ-Traces of Labelling Experiments 16

7. Crystallographic Data 17

8. References 27

1. Cellular Re Concentration Determined by ICP-MS

Table S1: Quantification of the rhenium levels in the nucleus, the mitochondria and the total cell in HT29 cells treated with the complexes. Values are given in ng/106 cells ± SD.

	Control	$(NH_4)[ReO_4]$	[**2**]$^+$	[**3**]$^+$	[**4**]$^{4+}$	[**6**]$^+$	[**7**]$^+$
Nucleus	0.13 ± 0.08	0.15 ± 0.00	43 ± 2.9	23 ± 4.2	21 ± 0.00	129 ± 9.9	29 ± 0.00
Mitochondria	0.06 ± 0.03	0.04 ± 0.00	12.5 ± 2.1	6.7 ± 0.99	5.6 ± 0.49	38 ± 22	8.1 ± 0.92
Total cell	0.21 ± 0.08	0.13 ± 0.03	233 ± 15	69 ± 5.7	73.3 ± 0.71	1482 ± 31	62 ± 7.1

2. Synthetic Procedures

General Materials and Methods

Starting materials **L1**,[1] [**1**]$^{+2}$ and [**5**]$^{+2,}$[3] were produced according to literature procedures. All chemicals were of reagent grade or higher, obtained from commercial sources (*Fluorochem*, *Alpha Aesar*, *Merck*, *Fluka* and *Fisher Scientific*) and used without further purification. Solvents were of p.a. grade or distilled prior to their use; H_2O was bidistilled. Deuterated NMR-solvents were purchased from *Armar Chemicals* or *Cambridge Isotope Laboratories*, *Inc. (UK).*

Radiation protection: ^{99m}Tc is a γ-emitter. Experimentation with ^{99m}Tc must be conducted in a licensed laboratory featuring appropriate radiation protection infrastructure such as shielding or dosimetry.

pH indicators were *Merck* indicator paper pH 1–14 (universal indicator).

NMR: *Bruker AV2-400* (400 MHz) or *Bruker AV2-500* (500 MHz); in deuterated solvents at 300 K; chemical shifts (δ) in ppm relative to residual CD_3CN solvent resonances (^{1}H 1.95 ppm, ^{13}C 0.45 ppm); coupling constants (J) in Hz. Signal assignments are based on 2D-NMR correlation experiments.

FT-IR: Jasco FT/IR-4200 (*JASCO International Co.,* Tokyo, Japan); wavenumbers in cm^{-1}. Samples were recorded as KBr pellets in an appropriate sample holder. Where *s* = strong, *m* = medium, *w* = weak signals.

HR-ESI-MS: *QExactive* (*Thermo Fisher Scientific*, Bremen, Germany) equipped with a heated ESI source connected to a *Dionex Ultimate 3000* UPLC system. Samples dissolved in MeOH, MeOH/CH_2Cl_2 3:1, MeOH/H_2O 1:1, DMSO/H_2O 1:10, or H_2O at ca. 50 µg mL^{-1} ; injection of 1 µL on–flow with an XRS auto-sampler (*CTC*, Zwingen, Switzerland)(mobile phase: MeOH + 0.1% HCOOH or CH_3CN/H_2O (2:8) + 0.1% HCOOH; flow rate 120 µL mL^{-1}); ion source parameters: spray voltage 3.0 kV, capillary temperature 280 °C, sheath gas 30 L min^{-1}, s-lens RF level 55.0; aux gas temperature 250°C; full scan MS in alternating (+)/(−)-ESI mode; mass ranges 80–1'200, 133–2'000, or 200–3'000 amu; resolution (full width half-maximum) 70'000; automatic gain control (AGC) target 3.00 106 ; maximum allowed ion transfer time (IT) 30 ms; mass calibration <2 ppm accuracy for *m/z* 130.06619–1621.96509 in (+)-ESI with *Pierce*® ESI calibration solutions (*Thermo Fisher Scientific*, Rockford, USA); lock masses: ubiquitous erucamide (*m/z* 338.34174, (+)-ESI).

UHPLC-ESI-MS: Samples (2 µl injection) were analyzed with a *Vanquish*™ *Horizon UHPLC* System (*Thermo Fisher Scientific*, Waltham, USA) connected to a *Vanquish*™ eλ detector and ISQ-EM ESI mass spectrometer (*Thermo Fisher Scientific*, Waltham, USA), operated in positive or negative mode; scan range *m/z* 200–1500. Separation was performed with an *Acquity BEH C18* HPLC column (1.7 µm particle size, 2x100 mm, *Waters*) kept at 40 °C. The mobile phase consisted of A: H_2O + 0.1% HCOOH and B: CH_3CN + 0.1% HCOOH. UV spectra were recorded between 190 and 670 nm at a 4 nm resolution and at 5 Hz. The mass spectrometer was operated in the positive (negative) electrospray ionization mode

at 3000 V (-2’000 V) capillary voltage with a N_2 sheath gas pressure of 41.9 psi, auxiliar gas pressure of 5.5 psi and sweep gas pressure of 0.1 psi. Vaporizer temperature was 238 °C. Spectra were acquired in the mass range from m/z 150 to 2’000 collecting two points s^{-1}.

ICP-MS: CP-MS measurements were performed with an Agilent QQQ 8800 Triple quad ICP-MS spectrometer, equipped with a standard x-lens setting, nickel cones and a “micro-mist” quartz nebulizer. The feed was 0.1 ml/min, the RF power 1550 W. Tune settings were based on the Agilent General Purpose method and only slightly modified by an autotune procedure using an Agilent 1 ppb tuning solution containing Li, Y, Ce and Tl. Values are reported as the average of 30 sweeps x 3 replicates. Elements were measured in a “helium-mode”. The name is referring to the gas in the reaction cell. All solutions were prepared from 60% HNO_3 (Merck 1.01518.1000 ultrapure), 30% HCl (Merck 1.01514.1000 ultrapure), or aqua regia (1:3 mixture of 60% HNO_3 and 30% HCl 1:3, ultrapure) and 18.2 MΩ Millipore water. Elements were measured against a serial dilution made with the following standards: Copper: Merck 1.70313.0100 in 2% HNO_3, Rhenium: Merck 1.70344.0100 in HO. Indium: Merck 1.70324.0100 in 2% HNO_3 was used as internal standard

Elemental Analysis: For C, H and N: *Leco Truespec*. Detection of analyte gasses over infra red (C as CO_2 and H as H_2O) and thermal conductivity (N as N_2). Samples containing S: *HEKAtech EuroVector*; detection via thermal conductivity.

Preparative HPLC: *Shimadzu Prominence Modular HPLC system*, comprised of a *CBM-40* controller module, *SPD-40* cell unit, *LC-20AP* binary pump module and an *FCV-200AL* quaternary valve, using a *Dr. Maisch Reprosil* C18 100-7 (40 x 250 mm) column. HPLC solvents were *dd*H_2O (0.1 vol% trifluoroacetic acid buffer) (solvent A) and HPLC grade acetonitrile (0.1 *vol*% trifluoroacetic acid buffer) (solvent B) and HPLC grad MeOH (0.1 *vol*% trifluoroacetic acid buffer) (Solvent C). The flow rate was 40 mL min^{-1}.

X-ray crystallography

Single crystal X-ray diffraction data were collected at 160.0(1) K on a Rigaku OD Synergy/Hypix diffractometer (**[6](PF$_6$)** and **[4](ReO$_4$)$_4$.(H$_2$O)$_2$**) or on a Rigaku OD Supernova/Atlas diffractometer (**[2](H)(PF$_6$)$_2$** and **[JOS-181b](ReO$_4$)$_4$.(H$_2$O)$_2$**) or on a Rigaku Synergy/Pilatus diffractometer (**[3]Cl.(H$_2$O)$_2$**) using the copper X-ray radiation (l = 1.54184 Å) from a dual wavelength X-ray source and an Oxford Instruments Cryojet XL cooler. The selected suitable single crystals were covered with oil (Infineum V8512, formerly known as Paratone N), mounted on a flexible nylon loop attached to a CrystalCap Magnetic™ pin (Hampton Research) and transferred to the goniometer head inside the diffractometer. Pre-experiment, data collection, data reduction and analytical absorption correction[4] were performed with the program suite *CrysAlisPro*.[5] Using Olex2,[6] the structure was solved with the SHELXT[7] small molecule structure solution program and refined with the SHELXL 2018/3 program package[8] by full-matrix least-squares minimization on F^2. PLATON[9] was used to check the result of the X-ray analysis. CCDC entries 2369413 (**[2](H)(PF$_6$)$_2$**), 2369414 (**[3]Cl.(H$_2$O)$_2$**), 2369415 (**[4](ReO$_4$)$_4$.(H$_2$O)$_2$**), 2369416 (**[6](PF$_6$)**) and 2369417 (**[JOS-181b](ReO$_4$)$_4$.(H$_2$O)$_2$)** contain the supplementary crystallographic data for this paper. These data are provided free of charge www.ccdc.cam.ac.uk/structures.

The crystal structure of **[2](H)(PF$_6$)$_2$** was refined as a 2-component twin with scale factors of 0.2757(8) and 0.7243(8). The minor component is rotated by 179.98° around [0.00 0.00 1.00] (reciprocal space) or [0.16 0.00 0.99] (direct space). The HKLF5 file was generated by *CrysAlisPro*. The H atoms bound to the N atoms were placed in the positions indicated by a difference electron density map; their position was refined with a bond length restraint while the isotropic displacement parameter was fixed to a value equal to 1.2U_{eq} of its parent atom. In the crystal structure of **[3]Cl.(H$_2$O)$_2$**, the ions cocrystallized with solvent molecules of water in a ratio 1/1/2, respectively. In the crystal structure of

[4](ReO$_4$)$_4$.(H$_2$O)$_2$, the ions co-crystallized with solvent molecules of water in a ratio 1/4/2, respectively. The cations lie on center of inversions. The H atom bound to the N atoms was placed in the position indicated by a difference electron density map; its position was refined with a bond length restraint while the isotropic displacement parameter was freely refined. The crystal structure of **[6](PF$_6$)** was refined as a 2-component twin with scale factors of 0.3542(7) and 0.6458(7). The minor component is rotated by 179.93° around [0.98 -0.00 -0.22] (reciprocal space) or [1.00 -0.00 0.00] (direct space). The HKLF4 and HKLF5 files were generated by *CrysAlisPro*. The H atoms bound to the N atoms were placed in the positions indicated by a difference electron density map; their position was freely refined while the isotropic displacement parameter was fixed to a value equal to 1.2U_{eq} of its parent atom. The PF_6^- counterion is partially disordered over two sets of positions with site-occupancy factors of 0.483(8) and 0.517(8). Similarity restraints were applied to the P-F bond lengths while the corresponding F atoms were restrained to have similar atomic displacements. In the crystal structure of **[JOS-181b](ReO$_4$)$_4$.(H$_2$O)$_2$)**, the asymmetric unit contain two different cations, $C_{27}H_{24}ClCuN_4ORe^+$ and $C_{27}H_{22}Cl_2CuN_4Re^{2+}$, two different anions, PF_6^- and Cl^-, and solvent molecules of water (in a ratio 1/1/2/1/2, respectively). The H atoms bound to the N and O atoms were placed in the positions indicated by a difference electron density map; their position was refined with a bond length restraint while the isotropic displacement parameter was fixed to a value equal to 1.2U_{eq}(N) and 1.5U_{eq}(O). One of the two PF_6^- counterions is partially disordered over two sets of positions with site-occupancy factors of 0.418(6) and 0.582(6). Similarity restraints were applied to the P-F bond lengths while the corresponding F atoms were restrained to have similar atomic displacements.

Cell Culture: The RPE-1 cell line was cultured in DMEM/F12 medium (Gibco). The HT29 cell line was cultured in McCoy medium (Gibco). The A549 cell line cell line was cultured in F12K medium (Gibco)/ All cell lines were complemented with 10% of fetal calf serum (Gibco) and 100 U.mL-1 penicillin-streptomycin mixture (Gibco) and maintained in a humidified atmosphere at 37 °C and 5% CO_2.

Cell Viability Assay using a 2D Cellular Model: The cytotoxicity of the compounds was assessed by a fluorometric cell viability assay using Resazurin (Acros Organics). Briefly, cells were seeded in triplicate in 96-well plates at a 4×10^3 cells/well density in 100 µL Gibco McCoy's 5A (1X) + GlutaMAX (Modified Medium) with 10 % FBS and 1% Penicillin/Streptomycin. After 24 h, cells were treated with increasing concentrations of the compounds and cisplatin. Dilutions for the compounds were prepared from 10 mM stock in DMSO which were diluted to 100 - 0,01 µM with medium. After 48 h of incubation, the medium was removed and 100 µL of complete medium containing resazurin (0.2 mg.mL^{-1} final concentration) was added. After 4 h of incubation at 37 °C, the fluorescence signal of the resorufin product was read (ex 540 nm, em 590 nm) in an Infinite 200 PRO Microplate Reader from TECAN. IC_{50} values were then calculated using the GraphPad Prism software.

Mito stress assay: 10^4 HT29 cells/well was seeded in a Seahorse XF Cell 96-well culture microplate using 80 µL of Gibco McCoy's 5A (1X) + GlutaMAX (Modified Medium) supplemented with 10% FBS and and 1% Penicillin/Streptomycin incubated for 24 h at 37 °C with 5% CO_2. Dilutions of stock solutions of the compounds in DMSO were done in medium to reach concentrations corresponding to the IC_{25}. Cisplatin was dissolved in an aqueous NaCl solution to reach a concentration corresponding to the IC_{25}. Medium was replaced with the compounds solutions and cells were incubated for 4 h. Medium was removed, and the cells were washed very carefully with Agilent Seahorse XF RPMI Medium three times. The Mito stress assay was run in an Agilent Seahorse XFe96 instrument at 37 °C using multiple inhibitors, i.e., ATP synthase inhibitor (oligomycin, 1 µM), proton gradient, mitochondrial membrane potential collapsing agent (FCCP, 1 µM), and mitochondrial respiratory complex I and III inhibitors (rotenone, 1 µM and antimycin A, 1 µM, respectively). At the end of the run, the cells were fixed using a 4% p-

formaldehyde solution and stained with Hoechst 33342. Each well was imaged in a Cytation 5 Cell Imaging Multimode Reader, BioTek using a 10X objective lens. Finally, the number of cells from each image was calculated by using Gen5 software and by utilizing the cell count and the data were normalized against the same cell number.

ICP-MS Cellular uptake studies: HT29 cells were seeded in Thermo Scientific 15 cm diameter Petri dishes with Gibco McCoy's 5A (1X) + GlutaMAX (Modified Medium) supplemented with 10% FBS and and 1% Penicillin/Streptomycin until the cells reach confluence. They were treated with 10 µM of the corresponding compounds diluted in the cell culture medium from a 10 mM DMSO stock solution. After 24 h for [**2**]$(TFA)_2$, [**3**]ReO_4, [**4**]$(ReO_4)_4$ and [**7**]Cl, and 4 h for[**6**]$(TFA)_3$, cells were trypsinated and counted. Fractionation of the cells and recovery of the mitochondrial, nuclear and total cell contents was performed using a Thermo Fisher NE-PER Nuclear and Cytoplasmic Extraction Reagents and Mitochondria Isolation Kit for Cultured cells kits. For total cell and nuclear contents extraction, $2x10^6$ cells were used. For mitochondrial contents extraction, $25x10^6$ cells were used. All samples were prepared in duplicates. After extraction, ICP-MS samples were prepared as follows: samples were digested using 70% nitric acid (0.5 mL, 70 °C, overnight) and then further diluted 1:100 (1% HCl solution in MQ water) analyzed using ICP-MS. All ICP-MS measurements were performed on a Agilent 7900 Quadrupole ICP-MS located at the Institut de Physique du Globe de Paris (France). The monitored isotopes are 99 and 101 Ru. Daily, before the analytical sequence, an indium internal standard was injected after inline mixing with the samples to correct for signal drift and matrix effects. A set of calibration standards was analyzed to confirm and model (through simple linear regression) the linear relationship between signal and concentration. The model was then used to convert measured sample counts to concentrations. The uncertainties were calculated using error propagation equations and considering the combination of standard deviation on the repeated consecutive signal acquisitions (n = 3), internal standard ratio and blank subtraction. The non-linear term (internal standard ratio) was linearized using a first-order Taylor series expansion to simplify error propagation. The amount of metal detected in the cell samples was transformed from ppb to µg of metal. Data were subsequently normalized to the number of cells and expressed as nanograms of metal/10^6 cells.

Reaction Schemes

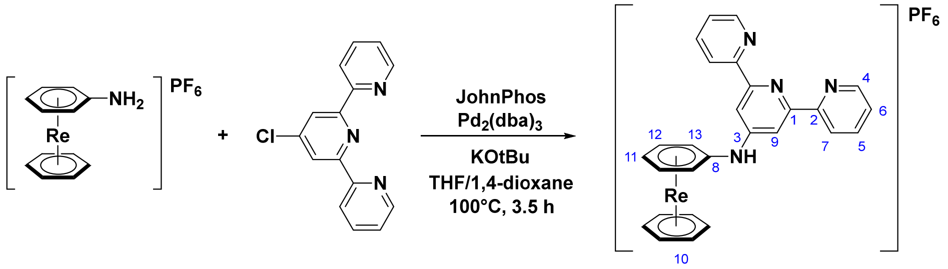

[1]PF_6 **4'-chloroterpy** **[2]**PF_6 (>95%)

Scheme S1: **[2]**PF_6

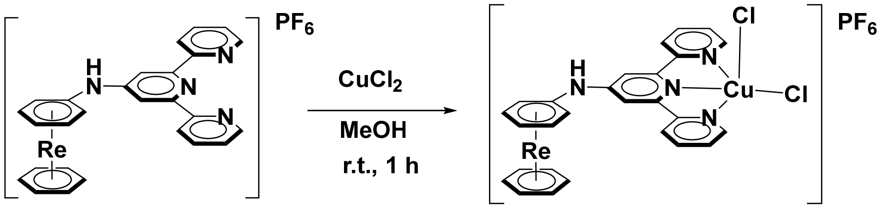

[2]PF_6 **[3]**PF_6 (85%)

Scheme S2: **[3]**PF_6

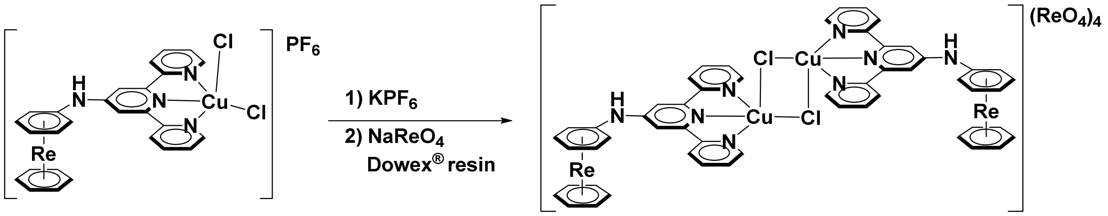

[3]PF_6 **[4]**$(ReO_4)_4$ (58%)

Scheme S3: **[4]**$(ReO_4)_4$

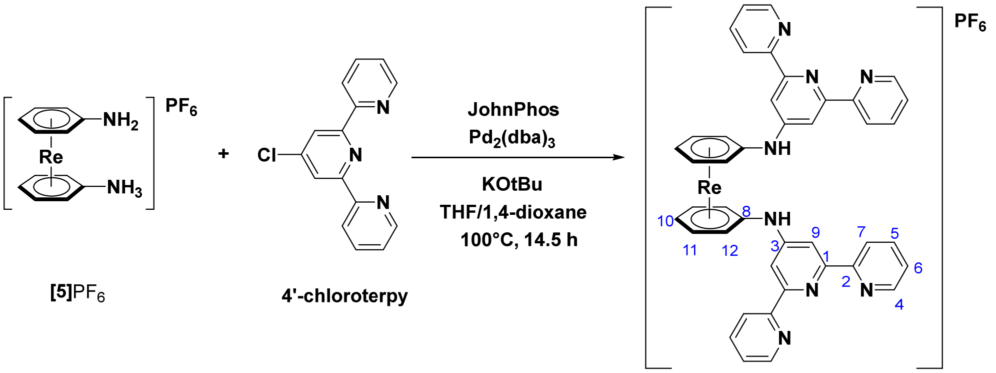

[5]PF_6 **4'-chloroterpy** **[6]**PF_6 (70%)

Scheme S4: **[6]**PF_6

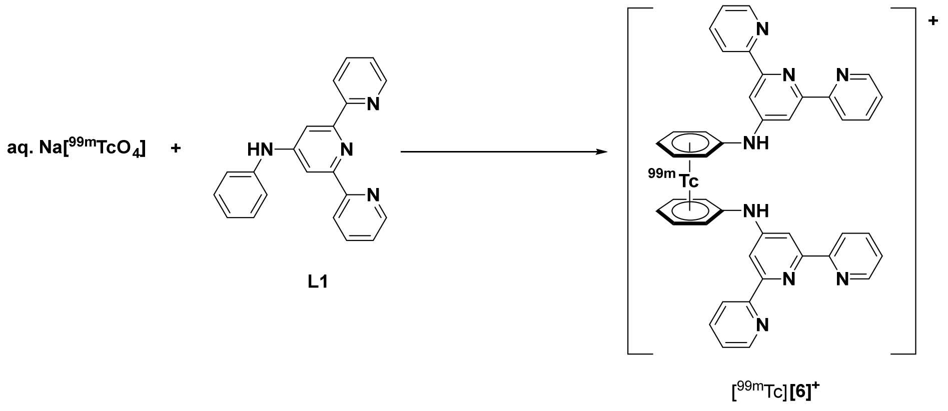

Scheme S5: [^{99m}Tc]**[6]**$^+$

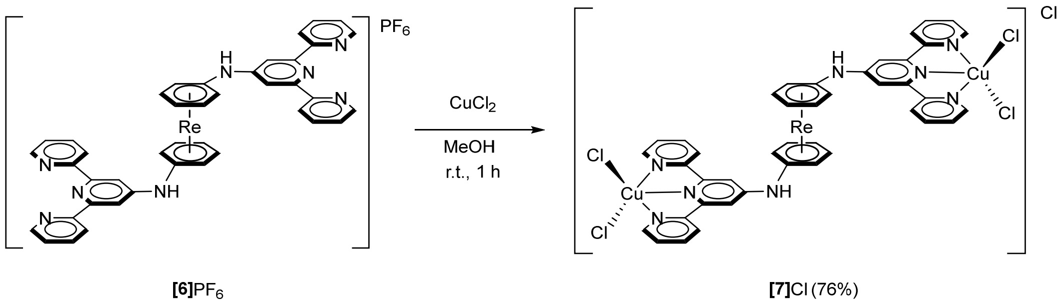

Scheme S6: **[7]**Cl

3. NMR Data

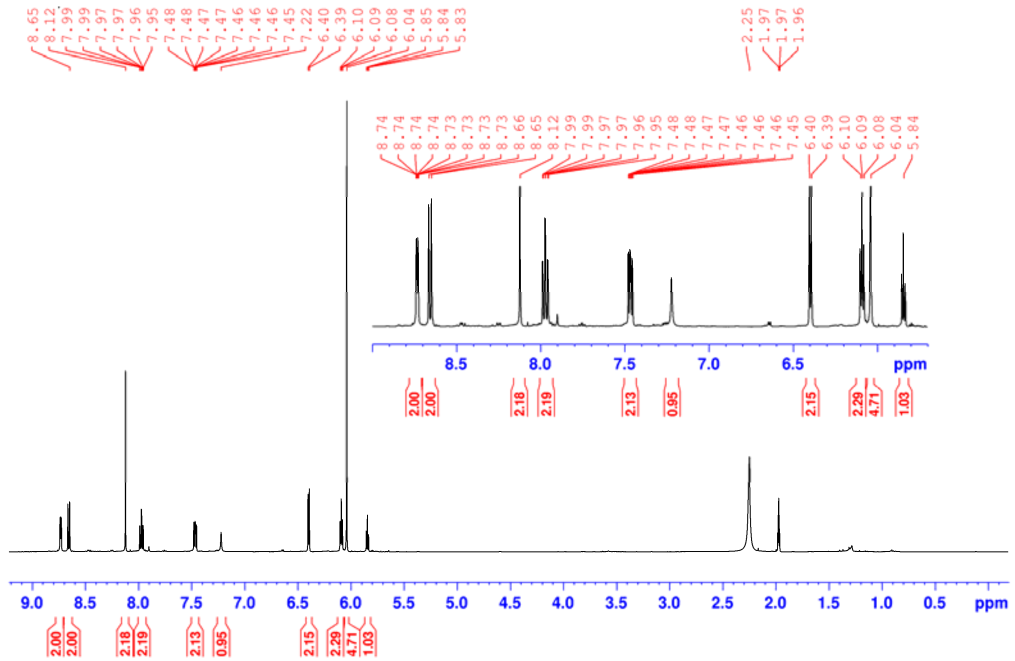

Figure S 1: ^{1}H NMR spectrum of **[2]**PF_6 in CH_3CN at 500 MHz.

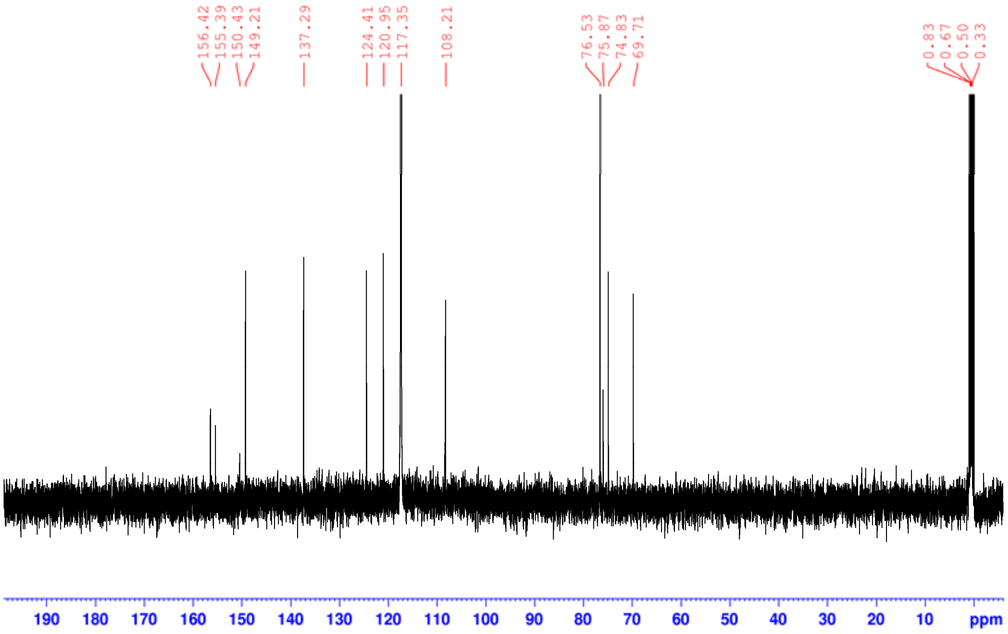

Figure S 2: ^{13}C NMR spectrum of **[2]**PF_6 in CH_3CN at 126 MHz.

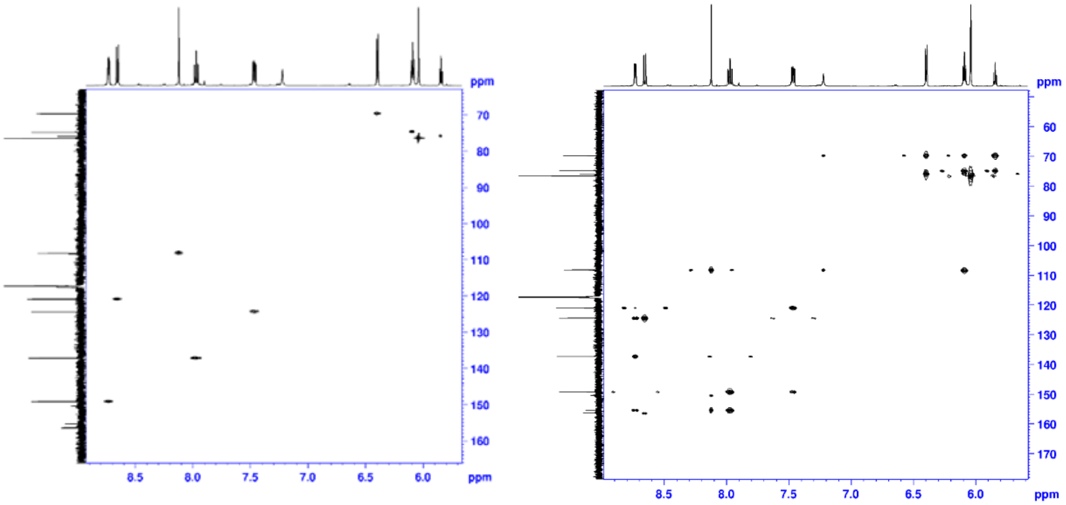

Figure S 3: HSQC (left) and HMBC (right) spectra of **[2]**PF_6 in CH_3CN at 500 MHz.

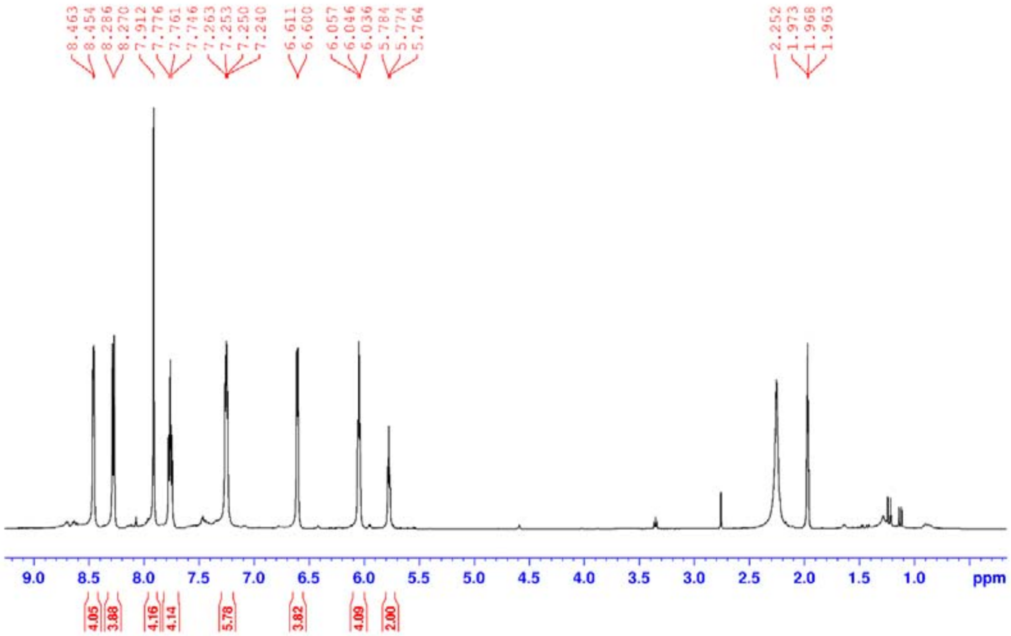

Figure S 4: ^{1}H NMR spectrum of **[6]**PF_6 in CH_3CN at 500 MHz.

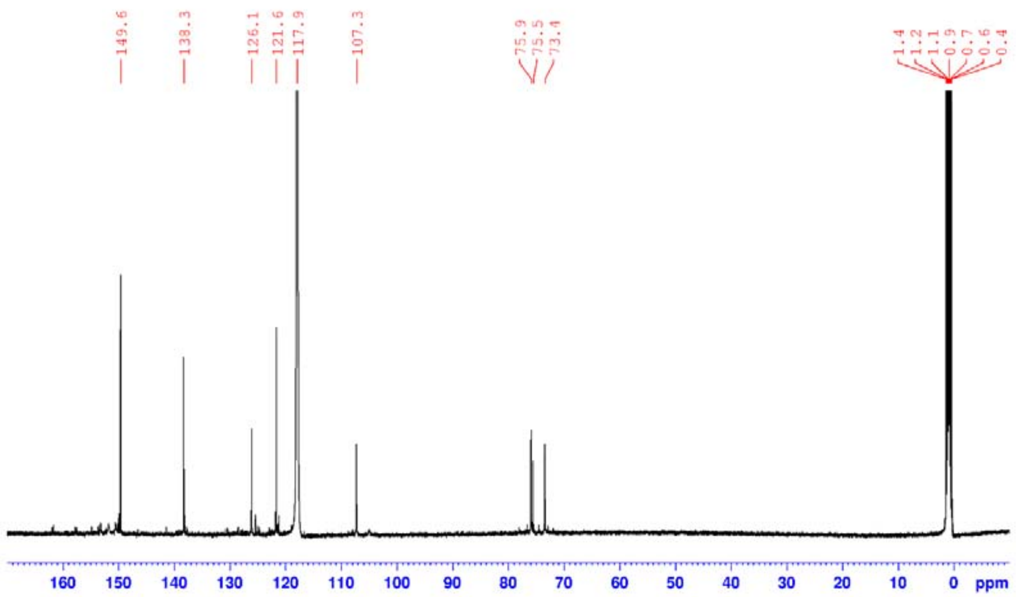

Figure S 5: ^{13}C NMR spectrum of **[6]**PF_6 in CH_3CN at 126 MHz.

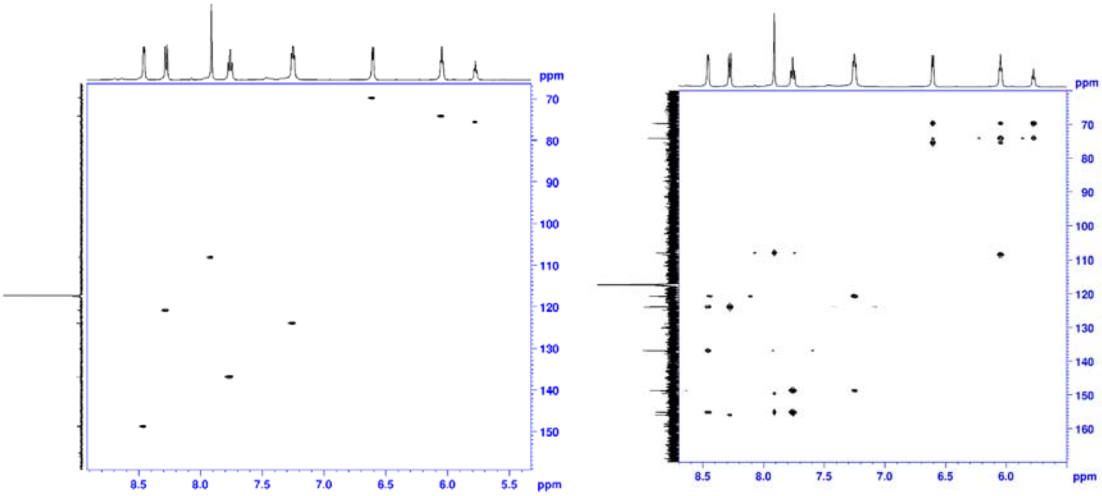

Figure S 6: HSQC (left) and HMBC (right) spectra of **[6]**PF_6 in CH_3CN.

4. FT-IR Data

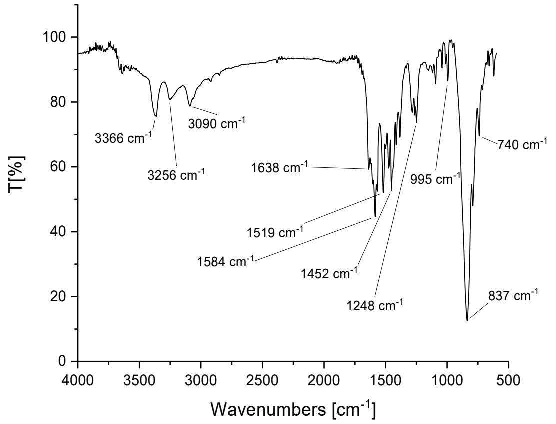

Figure S 7: FT-IR spectrum of **[2]**PF_6

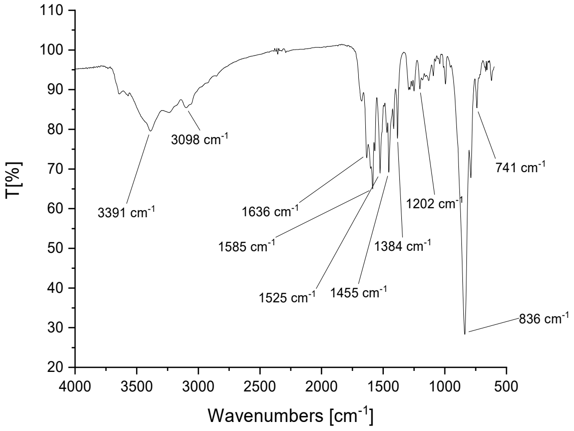

Figure S 8: FT-IR spectrum of **[6]**PF_6

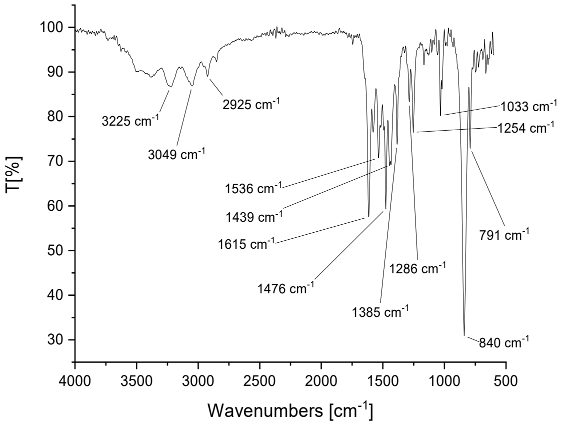

Figure S 9: FT-IR spectrum of **[3]**PF_6

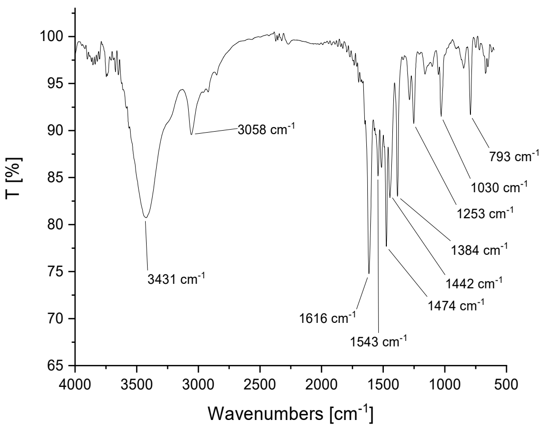

Figure S 10: FT-IR spectrum of **[4]**(ReO_4)$_4$.

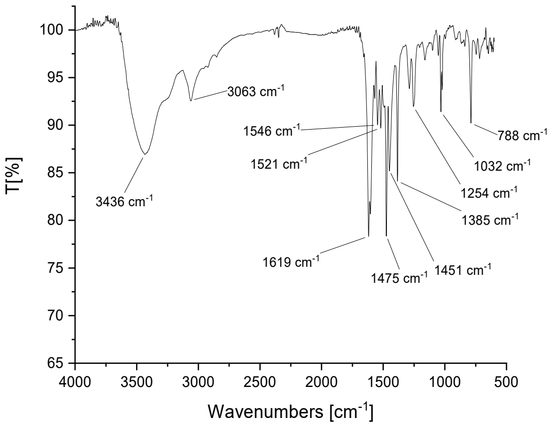

Figure S 11: FT-IR spectrum of **[7]**(ReO_4)$_4$.

5. HR-ESI-MS Data

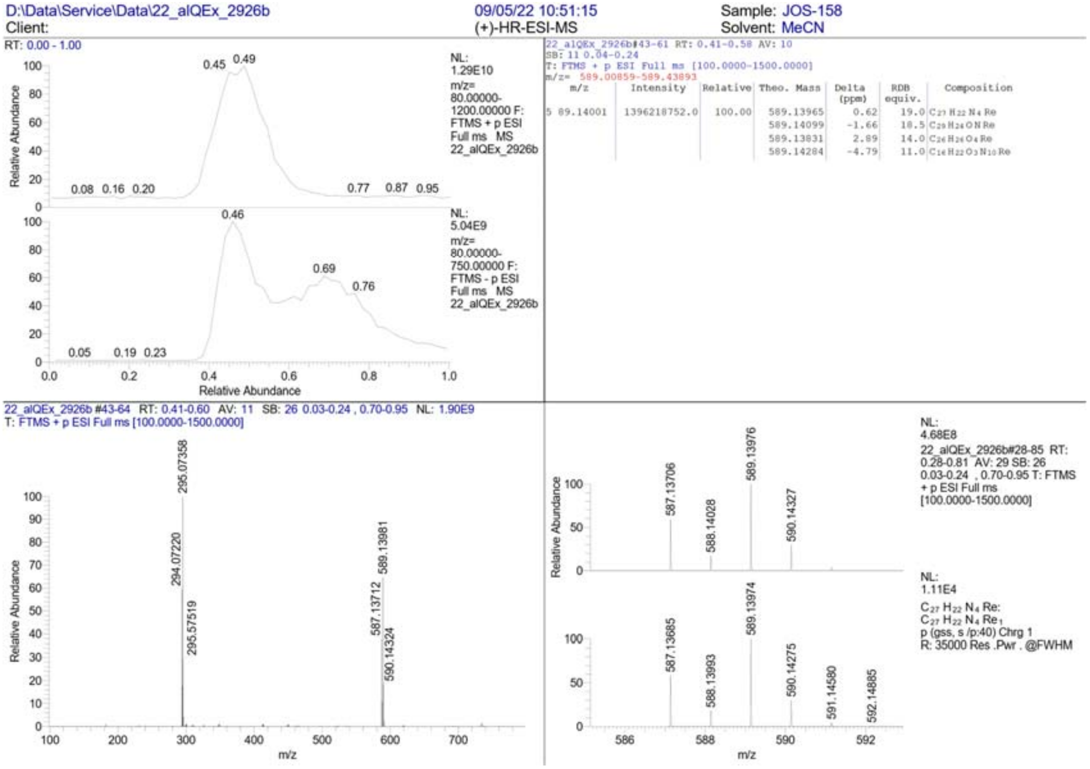

Figure S 12: HR-ESI-MS of **[2]**$^{+}$.

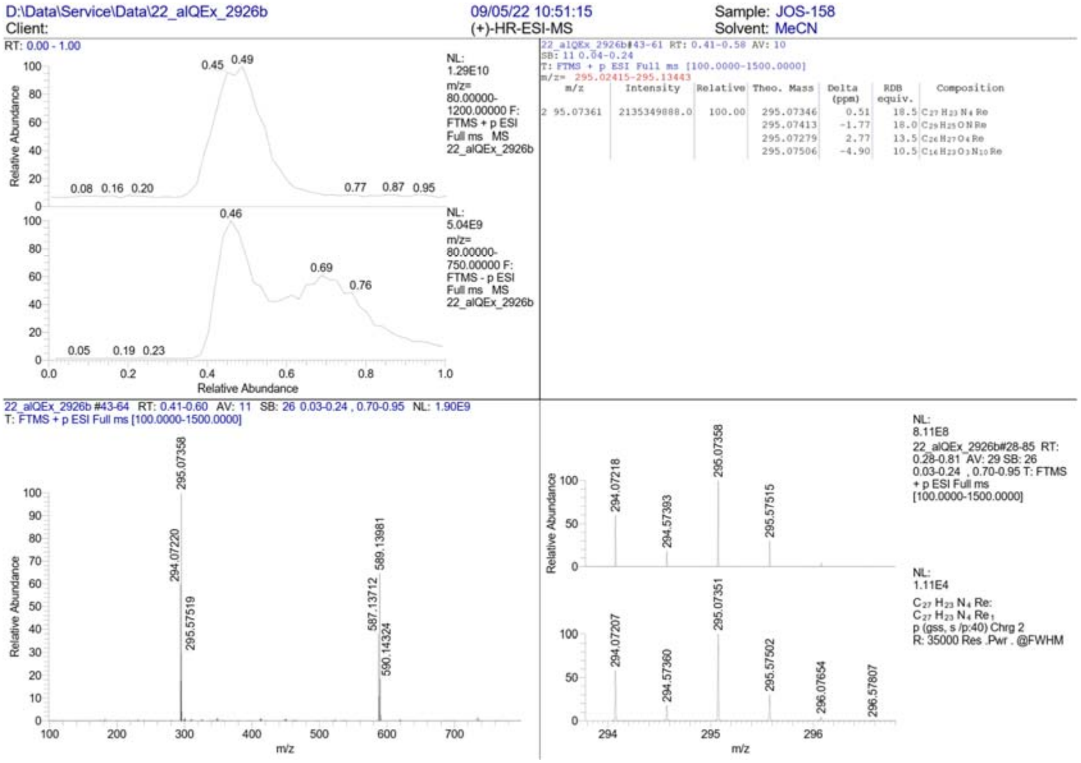

Figure S 13: HR-ESI-MS of [**[2]**+H]$^{2+}$.

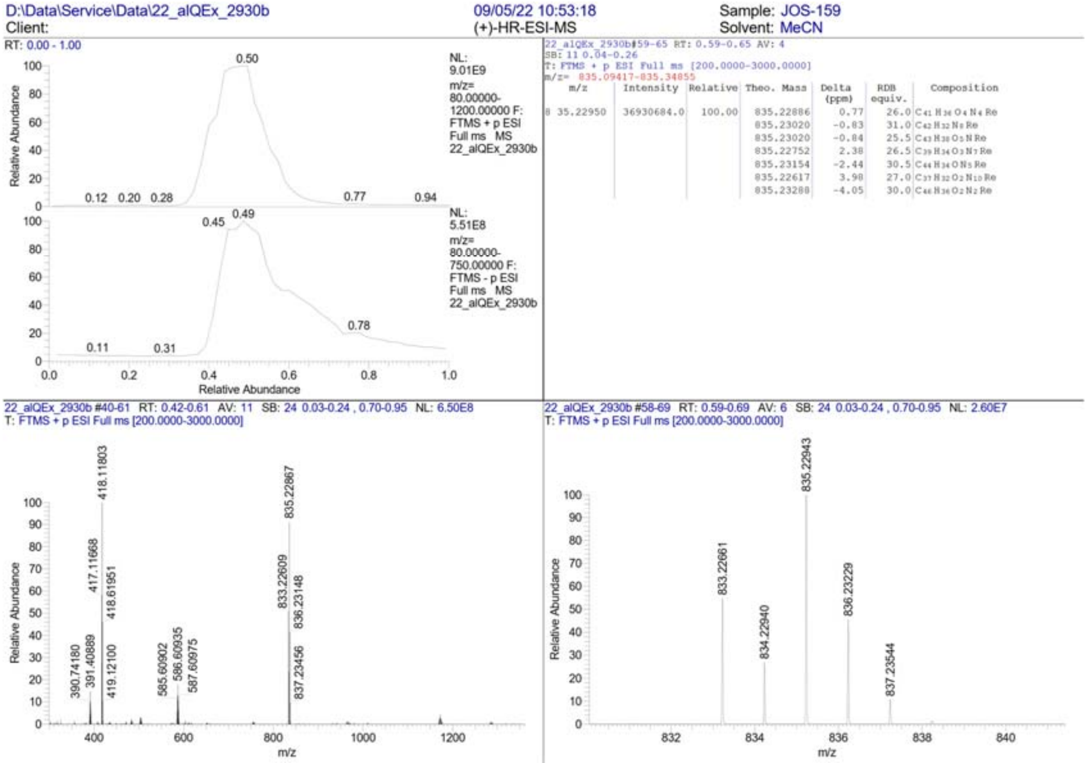

Figure S 14: HR-ESI-MS of **[6]**$^+$.

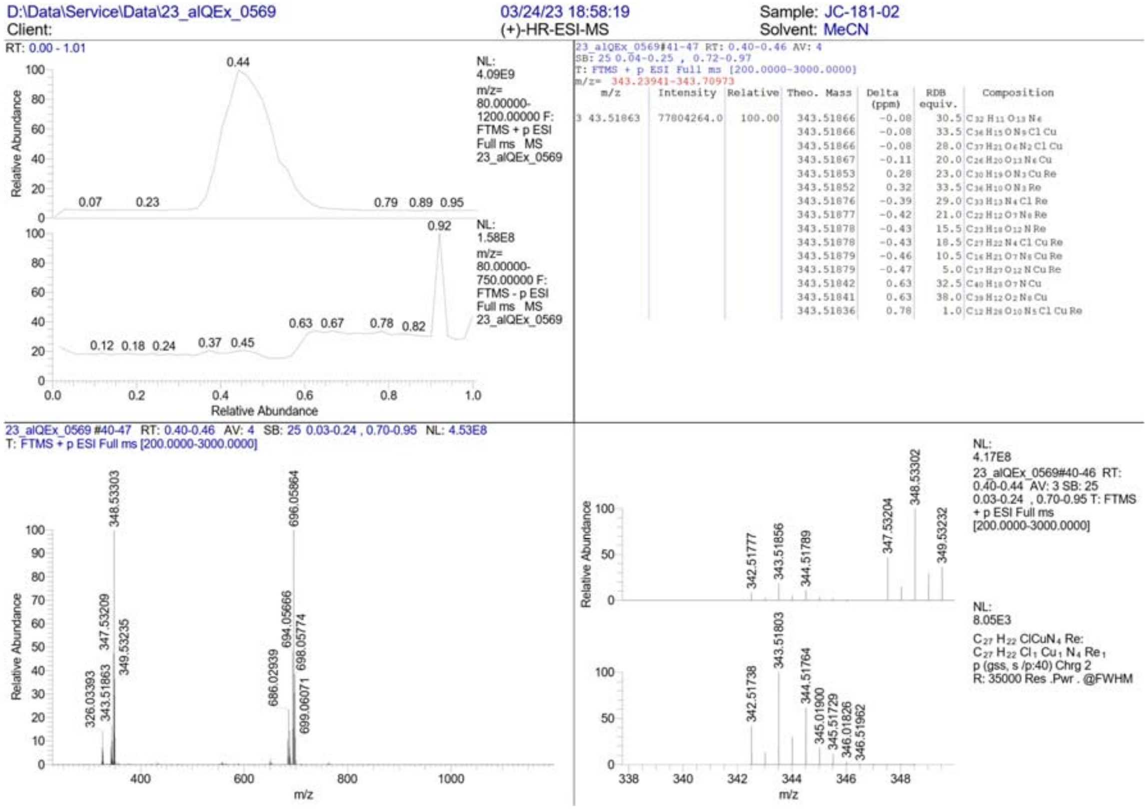

Figure S 15: HR-ESI-MS of **[3]**$^+$ as $[M-Cl]^{2+}$.

HR-ESI Report

Analysis Info

Analysis Name	D:\Data\UZH_Data\Data_2023\Service\Data\23_alhres_0148.d	Acquisition Date	4/14/2023 10:24:03 AM
Method	Service_Syringe_Pump_High_Mass_Range_pos.m	Operator	Demo User
Sample Name	JC-182-03	Instrument	timsTOF Pro 1854399.00195
Comment	Solvent: MeCN<br>Client: Csucker		

Acquisition Parameter

Source Type	ESI	Ion Polarity	Positive	Set Nebulizer	0.4 Bar
Focus	Not active			Set Dry Heater	200 °C
Scan Begin	50 m/z	Set Capillary	4500 V	Set Dry Gas	3.5 l/min
Scan End	3000 m/z	Set End Plate Offset	-500 V	Set Divert Valve	Source

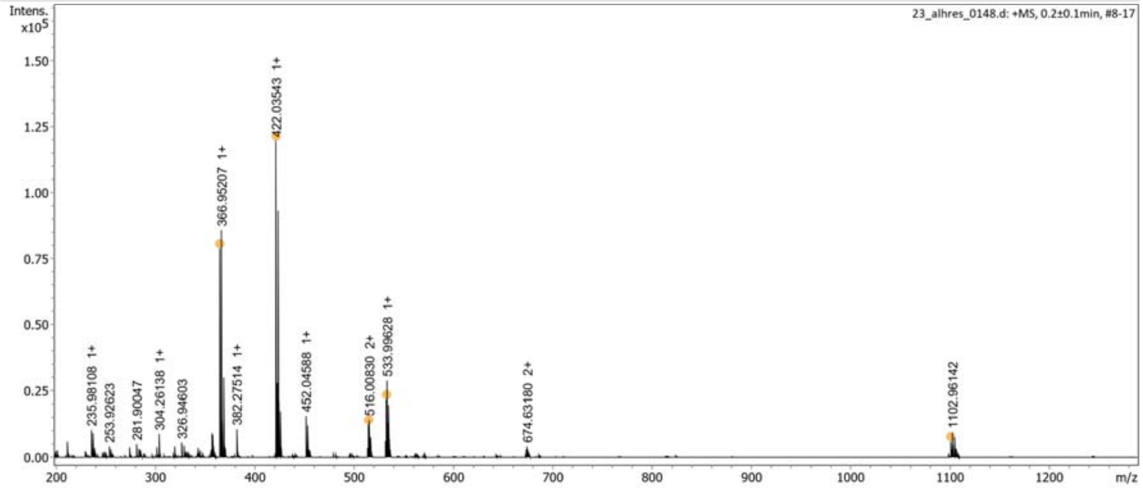

Bruker Compass DataAnalysis 5.3 printed: 4/14/2023 10:43:42 AM by: demo 1 of 1

HR-ESI Report

Meas. m/z	#	Ion Formula	Score	m/z	err [mDa]	err [ppm]	mSigma	rdb	e⁻ Conf
101.00301	1	C2H6NaOS	100.00	101.00316	0.14	1.43	3.4	2.0	even
179.01728	1	C2H7N6S2	88.19	179.01681	-0.47	-2.64	3.1	7.0	even
	2	C4H12NaO2S2	100.00	179.01709	-0.19	-1.07	3.2	3.0	even
364.95446	1	C15H10Cl2CuN3	100.00	364.95423	-0.23	-0.64	8.0	18.0	even
	2	C17H12Cl2CuO	58.79	364.95557	1.11	3.04	12.5	17.5	odd
422.03543	1	C21H16ClCuN4	100.00	422.03540	-0.04	-0.08	11.3	19.0	even
	2	C20H20ClCuO4	48.15	422.03406	-1.37	-3.25	13.2	14.0	even
515.00859	1	C42H31Cl2Cu2N8Re	100.00	515.00936	-0.35	-0.68	20.4	40.0	even
532.99670	1	C42H32Cl3Cu2N8Re	100.00	532.99770	-0.27	-0.51	8.9	42.0	even
1100.96256	1	C42H32Cl4Cu2N8Re	100.00	1100.96480	-0.55	-0.50	43.2	44.0	even

Automatical internal calibration

Date:	4/14/2023 10:26:30 AM
Polarity:	Positive
Reference mass list:	ESI: Tuning Mix ES-TOF (ESI)
Calibration mode:	HPC Calibration
Standard deviation:	1.867 ppm

Reference m/z	Resulting m/z	Intensity	Error [ppm]
118.08625	118.08595	27822	-2.610
322.04812	322.04832	7120	0.599
622.02896	622.02834	8271	-0.996
922.00980	922.00944	5895	-0.385
1221.99064	1221.99122	5081	0.475
1521.97148	1521.97166	2472	0.118
1821.95231	1821.95089	822	-0.783
2121.93315	2121.93487	566	0.813
2421.91399	2421.91354	191	-0.184
2721.89483	2721.89531	145	0.176

Bruker Compass DataAnalysis 5.3 printed: 4/14/2023 10:42:16 AM by: demo 2 of 2

Figure S 16: HR-ESI-MS of **[7]**$^+$.

6. HPLC UV/Vis and γ-Traces of Labelling Experiments

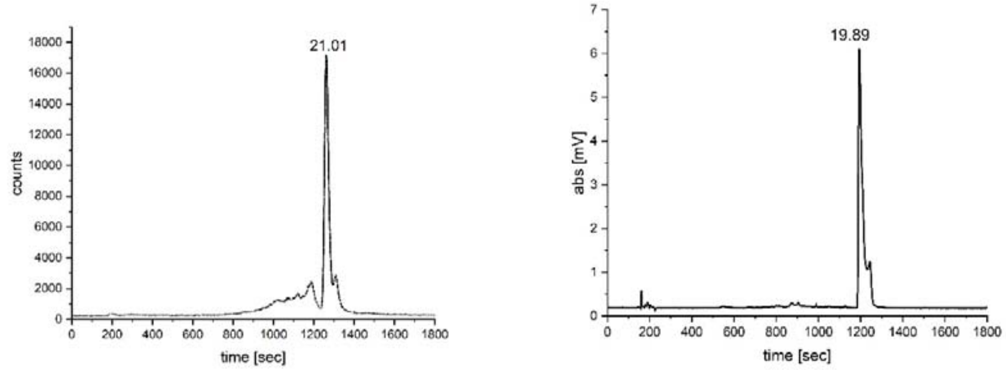

Figure S 17: HPLC γ-trace of the crude reaction mixture before purification (left) and corresponding UV/vis trace (right), showing the absorption peak of **L1**. Retention times might slightly vary due to ligand overload.

7. Crystallographic Data

Data of **[2]**(H)(PF_6)$_2$

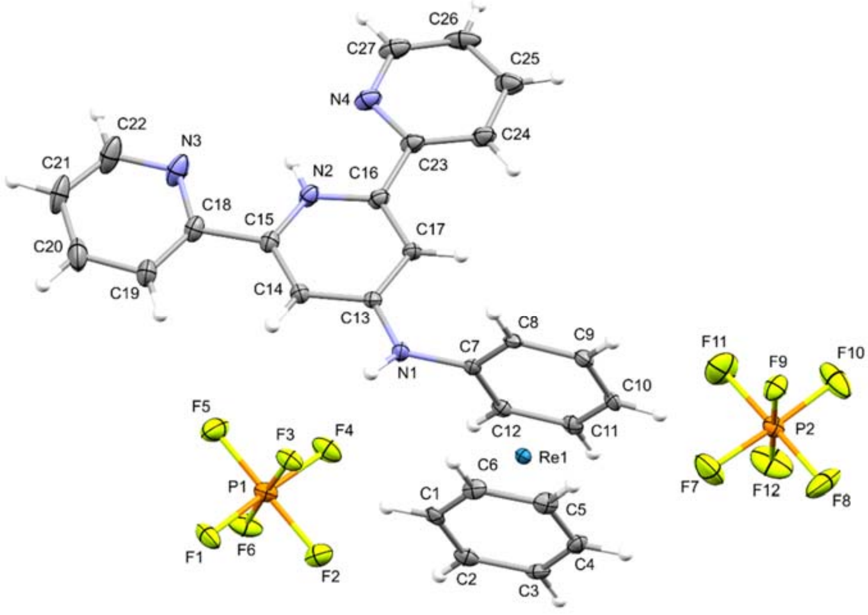

Figure S18: Ellipsoid displacement plot[10] of **[2]**(H)(PF_6)$_2$. Hydrogen atoms and counter ions were omitted for clarity. Thermal ellipsoids represent 50% probability.

Special features

Refined as a two-component twin with scale factors of 0.2757(8) and 0.7243(8). Component two is rotated by 179.98° around [0.00 0.00 1.00] (reciprocal space) or [0.16 0.00 0.99] (direct space). The HKLF5 file was generated by *CrysAlisPro*.[2]

Table S2: Tabulated values of selected bond lengths and angles of the crystallographic data of **[2]**(H)(PF_6)$_2$.

Selected bond lengths [Å]		Selected angles [°]	
N1-C7	1.424(7)	C7-N1.C13	124.2(5)
N1-C13	1.339(7)	C7-Re1-C1	103.6(2)
C15-C18	1.488(8)	N3-C18-C15	113.9(6)
C16-C23	1.481(9)	N2-C15-C28	115.0(5)
C7-Re1	2.215(5)		

Table S3: Crystal data and structure refinement for [2](H)(PF_6)$_2$.

Identification code	josh2609_tw
Empirical formula	$C_{27}H_{23}F_{12}N_4P_2Re$
Formula weight	879.63
Temperature/K	160(1)
Crystal system	monoclinic
Space group	$P2_1/c$
a/Å	9.68242(12)
b/Å	9.74690(11)
c/Å	31.3243(3)
α/°	90
β/°	92.8776(10)
γ/°	90
Volume/Å^3	2952.45(6)
Z	4
ρ_{calc}g/cm^3	1.979
μ/mm^{-1}	10.035
F(000)	1704.0
Crystal size/mm^3	0.12 × 0.09 × 0.03
Radiation	Cu Kα (λ = 1.54184)
2Θ range for data collection/°	9.146 to 136.702
Index ranges	-11 ≤ h ≤ 11, -11 ≤ k ≤ 11, -36 ≤ l ≤ 37
Reflections collected	7872
Independent reflections	7872 [R_{int} = ?, R_{sigma} = 0.0112]
Data/restraints/parameters	7872/2/422
Goodness-of-fit on F^2	1.147
Final R indexes [I>=2σ (I)]	R_1 = 0.0309, wR_2 = 0.1011
Final R indexes [all data]	R_1 = 0.0332, wR_2 = 0.1028
Largest diff. peak/hole / e Å^{-3}	0.74/-0.82
CDCC number	2369413

Data of **[6]**PF_6

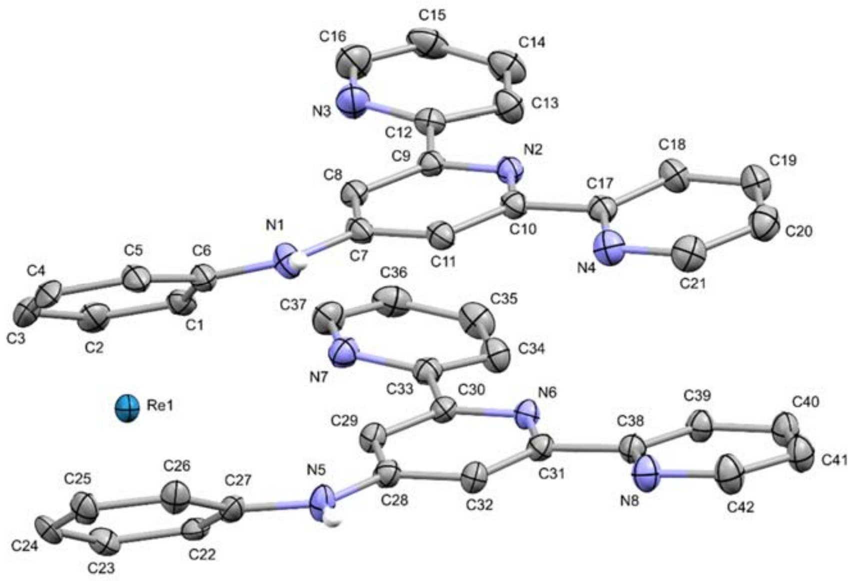

Figure S19 Ellipsoid displacement plot[10] of **[6]**PF_6. Hydrogen atoms and counter ions were omitted for clarity. Thermal ellipsoids represent 50% probability.

Table S4: Tabulated values of selected bond lengths and angles of the crystallographic data of **[6]**PF_6**.**

Selected bond lengths [Å]		Selected angles [°]	
N1-C6	1.389(7)	C6-N1-C7	129.6(5)
N1-C7	1.399(6)	C27-N5-C28	129.2(5)
N5-C27	1.405(6)	C6-Re1-C27	102.96(18)
N5-C28	1.379(6)	N2-C9-C12	115.6(4)
10-C17	1.486(7)	N2-C10-C17	115.7(4)
C9-C12	1.481(7)	N6-C30-C33	117.2(4)
C30-C33	1.487(7)	N6-C31-C38	115.9(4)
C31-C38	1.491(6)		

Table S5: Crystal data and structure refinement for [6]PF_6.

Identification code	josh2111_tw
Empirical formula	$C_{42}H_{32}F_6N_8PRe$
Formula weight	979.92
Temperature/K	160.0(1)
Crystal system	monoclinic
Space group	$P2_1/c$
a/Å	7.45120(10)
b/Å	28.1712(6)
c/Å	17.4358(3)
α/°	90
β/°	95.461(2)
γ/°	90
Volume/Å^3	3643.32(11)
Z	4
ρ_{calc}g/cm^3	1.787
μ/mm^{-1}	7.603
F(000)	1936.0
Crystal size/mm^3	0.14 × 0.04 × 0.02
Radiation	Cu Kα (λ = 1.54184)
2Θ range for data collection/°	5.98 to 160.448
Index ranges	-9 ≤ h ≤ 9, -35 ≤ k ≤ 35, -21 ≤ l ≤ 22
Reflections collected	14173
Independent reflections	14173 [R_{int} = 0.0231, R_{sigma} = 0.0159]
Data/restraints/parameters	14173/148/568
Goodness-of-fit on F^2	1.084
Final R indexes [I>=2σ (I)]	R_1 = 0.0384, wR_2 = 0.1026
Final R indexes [all data]	R_1 = 0.0428, wR_2 = 0.1053
Largest diff. peak/hole / e Å^{-3}	0.97/-1.23
CDCC number	2369416

Data of **[3]**Cl·2(H_2O)

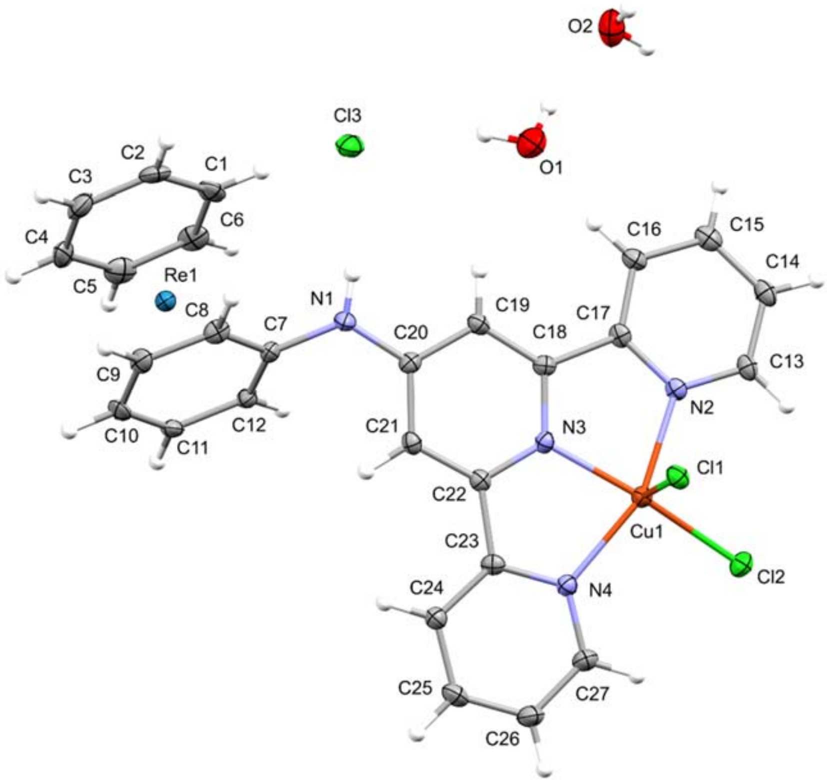

Figure S20: Ellipsoid displacement plot[10] of **[3]**$^{2+}$ of the crystal structure **[3]**Cl·(H_2O)$_2$. Counter ions were omitted for clarity. Thermal ellipsoids represent 50% probability.

Special features

The ions co-crystallized with solvent molecules of water in a ratio 1/1/2, respectively.

Table S6: Tabulated values of selected bond lengths and angles of the crystallographic data of **[3]**Cl·(H_2O)$_2$.

Selected bond lengths [Å]		Selected angles [°]	
N1-C7	1.412(7)	C7-N1-C20	124.3(4)
N1-C20	1.365(7)	N2-Cu1-N4	156.30(17)
N2-Cu1	2.040(4)	N3-Cu1-C2	158.41(14)
N3-Cu1	1.944(4)	N3-Cu1-C1	97.76(13)
N4-Cu1	2.037(5)	N2-Cu1-N3	78.95(18)
Cu1-Cl1	2.5176(15)	N3-Cu1-N4	79.15(18)
Cu1-Cl2	2.2625(15)	Cl1-Cu1-Cl2	103.82(6)
Re1-C1	2.217(5)		
Re1-C7	2.259(5)		

Table S7 Crystal data and structure refinement for [3]Cl·$(H_2O)_2$.

Identification code	josh1302
Empirical formula	$C_{27}H_{26}Cl_3CuN_4O_2Re$
Formula weight	794.61
Temperature/K	160.0(1)
Crystal system	monoclinic
Space group	$P2_1/c$
a/Å	9.1222(2)
b/Å	9.6159(2)
c/Å	30.1576(5)
α/°	90
β/°	91.437(2)
γ/°	90
Volume/Å^3	2644.54(9)
Z	4
ρ_{calc}g/cm^3	1.996
μ/mm^{-1}	12.877
F(000)	1548.0
Crystal size/mm^3	0.12 × 0.03 × 0.01
Radiation	Cu Kα (λ = 1.54184)
2Θ range for data collection/°	5.864 to 149.004
Index ranges	$-11 \le h \le 8$, $-11 \le k \le 11$, $-37 \le l \le 37$
Reflections collected	26951
Independent reflections	5364 [R_{int} = 0.0516, R_{sigma} = 0.0355]
Data/restraints/parameters	5364/5/359
Goodness-of-fit on F^2	1.075
Final R indexes [I>=2σ (I)]	R_1 = 0.0378, wR_2 = 0.0847
Final R indexes [all data]	R_1 = 0.0456, wR_2 = 0.0878
Largest diff. peak/hole / e Å^{-3}	1.55/-1.53
CCDC number	2369414

Data of **[3*][3]**$(ReO_4)_2$·$Cl(H_2O)_2$.

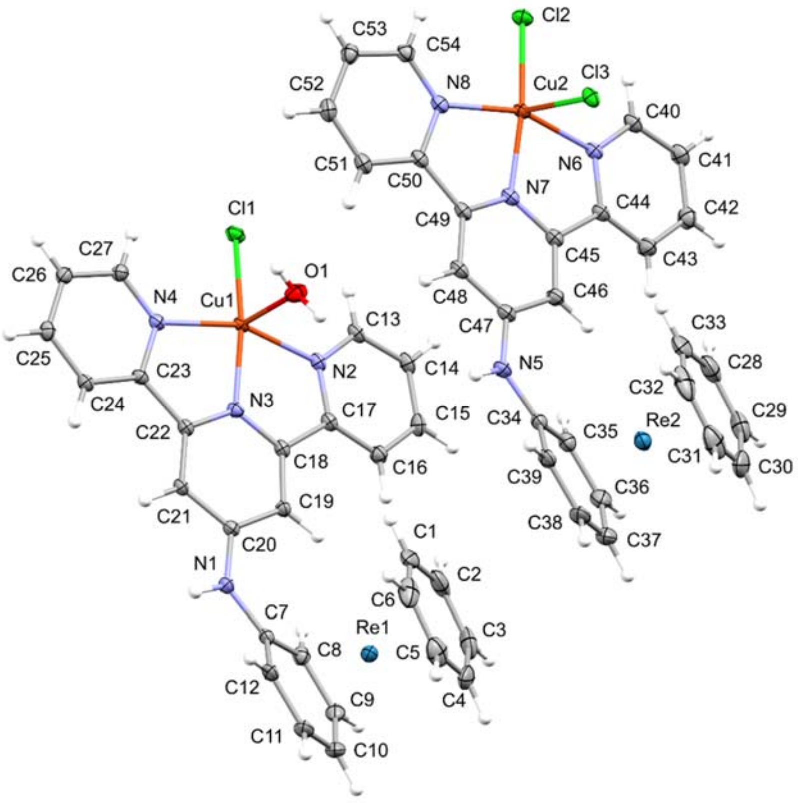

Figure S21: Ellipsoid displacement plot[10] of **[3*]**$^{2+}$ and **[3]**$^{+}$ of the crystal structure **[3*][3]**$(ReO_4)_2$·$Cl(H_2O)_2$. Counter ions were omitted for clarity. Thermal ellipsoids represent 50% probability.

Table S8: Tabulated values of selected bond lengths and angles of the crystallographic data of **[3*][3]**$(ReO_4)_2$·$Cl(H_2O)_2$.

Selected bond lengths [Å]		Selected angles [°]	
Cu1-Cl1	2.2105(7)	Cl1-Cu1-O1	98.84(7)
Cu1-O1	2.273(2)	N3-Cu1-O1	94.10(9)
Cu2-Cl2	2.2033(8)	Cl2-Cu2-Cl3	103.99(3)
Cu2-Cl3	2.6856(8)	N7-Cu2-Cl3	87.98(7)
Cu1-N2	2.042(2)	N2-Cu1-N4	158.26(9)
Cu1-N3	1.935(2)	N3-Cu1-N4	79.89(9)
Cu1-N4	2.041(2)	N6-Cu2-N8	158.96(10)
Cu2-N6	2.038(2)	N6-Cu2-N7	79.61(10)
Cu2-N7	1.925(2)	N7-Cu2-N8	80.30(10)
Cu2-N8	2.030(2)	C7-N1-C20	130.3(2)
N1-C7	1.386(4)	C34-N5-C47	129.8(3)
N1-C20	1.370(4)		
Re1-C7	2.313(3)		
Re2-C34	2.287(3)		

Table S9 Crystal data and structure refinement for [3*][3]$(ReO_4)_2 \cdot Cl(H_2O)_2$.

Identification code	josh1501
Empirical formula	$C_{54}H_{50}Cl_4Cu_2F_{12}N_8O_3P_2Re_2$
Formula weight	1790.24
Temperature/K	160.0(1)
Crystal system	triclinic
Space group	P-1
a/Å	10.4752(2)
b/Å	14.4269(2)
c/Å	20.3271(3)
α/°	94.8760(10)
β/°	93.4040(10)
γ/°	104.9670(10)
Volume/Å^3	2946.40(8)
Z	2
ρ_{calc}g/cm^3	2.018
μ/mm^{-1}	11.622
F(000)	1736.0
Crystal size/mm^3	0.11 × 0.06 × 0.02
Radiation	Cu Kα (λ = 1.54184)
2Θ range for data collection/°	4.378 to 148.994
Index ranges	-13 ≤ h ≤ 13, -18 ≤ k ≤ 15, -25 ≤ l ≤ 25
Reflections collected	66683
Independent reflections	12017 [R_{int} = 0.0331, R_{sigma} = 0.0236]
Data/restraints/parameters	12017/361/839
Goodness-of-fit on F^2	1.021
Final R indexes [I>=2σ (I)]	R_1 = 0.0260, wR_2 = 0.0649
Final R indexes [all data]	R_1 = 0.0296, wR_2 = 0.0675
Largest diff. peak/hole / e Å^{-3}	0.87/-0.98
CCDC number	2369417

Data of **[4](**ReO_4)$_4$·2(H_2O)

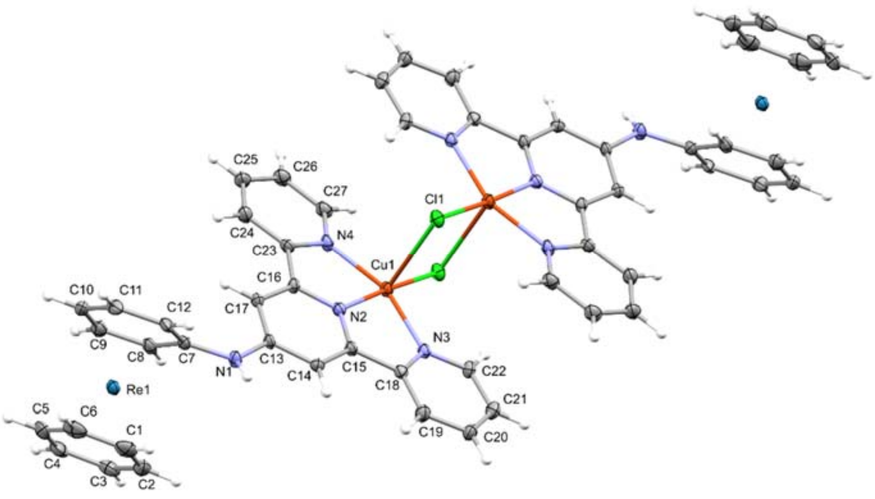

Figure S22: Ellipsoid displacement plot[10] of **[4]**$^{2+}$ of the crystal structure **[4]**(ReO_4)$_4$·(H_2O)$_2$. Counter ions were omitted for clarity.

Table S10: Tabulated values of selected bond lengths and angles of the crystallographic data of **[4]**(ReO_4)$_4$·(H_2O)$_2$.

Selected bond lengths [Å]		Selected angles [°]	
Cu1-Cl1	2.2302(15)	Cl1-Cu1-Cl1’	89.57(5)
Cu1-Cl1’	2.7123(17)	Cu1-Cl1-Cu1’	90.43(5)
Cu1-N2	1.922(5)	N3-Cu1-N2	79.7(2)
Cu1-N3	2.037(5)	N2-Cu1-N4	80.3(2)
Cu1-N4	2.024(5)	N3-Cu1-N4	158.23(19)
N1-C7	1.407(8)	N2-Cu1-Cl1’	174.25(16)
Re1-C7	2.276(6)		

Table S11: Crystal data and structure refinement for [4]$(ReO_4)_4 \cdot (H_2O)_2$

Identification code	josh0906
Empirical formula	$C_{54}H_{48}Cl_2Cu_2N_8O_{18}Re_6$
Formula weight	2412.18
Temperature/K	160.0(1)
Crystal system	triclinic
Space group	P-1
a/Å	6.3882(2)
b/Å	14.2466(2)
c/Å	17.5738(4)
α/°	70.439(2)
β/°	84.978(2)
γ/°	82.022(2)
Volume/Å^3	1491.05(6)
Z	1
ρ_{calc}g/cm^3	2.686
μ/mm^{-1}	25.375
F(000)	1114.0
Crystal size/mm^3	0.17 × 0.04 × 0.02
Radiation	Cu Kα (λ = 1.54184)
2Θ range for data collection/°	5.342 to 154.586
Index ranges	$-8 \le h \le 8$, $-12 \le k \le 18$, $-21 \le l \le 22$
Reflections collected	30665
Independent reflections	6262 [R_{int} = 0.0420, R_{sigma} = 0.0293]
Data/restraints/parameters	6262/1/414
Goodness-of-fit on F^2	1.087
Final R indexes [I>=2σ (I)]	R_1 = 0.0371, wR_2 = 0.1023
Final R indexes [all data]	R_1 = 0.0397, wR_2 = 0.1039
Largest diff. peak/hole / e Å^{-3}	2.98/-3.11
CCDC number	2369415

8. References

(1) Constable, E. C.; Housecroft, C. E.; Tao, Y. A Convenient Synthesis of Multitopic 2,2′:6′,2′′-Terpyridine Ligands. *Synthesis* **2004**, *2004* (06), 869-874.

(2) Nadeem, Q.; Battistin, F.; Blacque, O.; Alberto, R. Naphthalene Exchange in $[Re(\eta^6\text{-napht})_2]^+$ with Pharmaceuticals Leads to Highly Functionalized Sandwich Complexes $[M(\eta^6\text{-pharm})_2]^+$ (M=Re/^{99m}Tc). *Chem. Eur. J.* **2022**, *28* (5), e202103566.

(3) Csucker, J.; Jo, D. K.; Nadeem, Q.; Blacque, O.; Fox, T.; Braband, H.; Alberto, R. An isoindoline bridged $[M(\eta^6\text{-arene})_2]^+$ (M = Re, ^{99m}Tc) ansa-arenophane and its dinuclear macrocycles with axial chirality. *Dalton Trans.* **2022**, *51* (25), 9591-9595

(4) Clark, R. C.; Reid, J. S. The analytical calculation of absorption in multifaceted crystals. *Acta Cryst. A* **1995**, *51* (6), 887-897.

(5) CrysAlisPro (version 1.171.42.57a), R. O. D. L., Yarnton, Oxfordshire, England, 2022.

(6) Dolomanov, O. V.; Bourhis, L. J.; Gildea, R. J.; Howard, J. A. K.; Puschmann, H. OLEX2: a complete structure solution, refinement and analysis program. *J. Appl. Cryst.* **2009**, *42* (2), 339-341.

(7) Sheldrick, G. SHELXT - Integrated space-group and crystal-structure determination. *Acta Cryst. A* **2015**, *71* (1), 3-8.

(8) Sheldrick, G. Crystal structure refinement with SHELXL. *Acta Cryst. C* **2015**, *71* (1), 3-8.

(9) Spek, A. Structure validation in chemical crystallography. *Acta Cryst. D* **2009**, *65* (2), 148-155.

(10) Farrugia, L. ORTEP-3 for Windows - a version of ORTEP-III with a Graphical User Interface (GUI). *J. Appl. Cryst.* **1997**, *30* (5 Part 1), 565.
